# Phylodynamic and Epistatic Analysis of Coxsackievirus A24 and Its Variant

**DOI:** 10.3390/v16081267

**Published:** 2024-08-08

**Authors:** Chia-Chi Cheng, Pei-Huan Chu, Hui-Wen Huang, Guan-Ming Ke, Liang-Yin Ke, Pei-Yu Chu

**Affiliations:** 1 Department of Medical Laboratory Science and Biotechnology, College of Health Sciences, Kaohsiung Medical University, Kaohsiung 807378, Taiwan; armyemily900305@gmail.com; 2 Department of Cardiology, Wei-Gong Memorial Hospital, Miaoli 351498, Taiwan; skinchu0515@gmail.com; 3 Department of Anesthesiology, Kaohsiung Chang Gung Memorial Hospital, Chang Gung University College of Medicine, Kaohsiung 833401, Taiwan; july25@cgmh.org.tw; 4 Graduate Institute of Animal Vaccine Technology, College of Veterinary Medicine, National Pingtung University of Science and Technology, Pingtung 912301, Taiwan; kegm@mail.npust.edu.tw; 5 Drug Development and Value Creation Research Center, Kaohsiung Medical University, Kaohsiung 807378, Taiwan; 6 Center for Lipid Biosciences, Department of Medical Research, Kaohsiung Medical University Hospital, Kaohsiung 807377, Taiwan; 7 Department of Laboratory Medicine, Kaohsiung Medical University Hospital, Kaohsiung 807377, Taiwan

**Keywords:** Coxsackievirus A24 (CV-A24), Coxsackievirus A24 variant (CV-A24v), phylodynamics, genetic recombination, epistasis

## Abstract

Coxsackievirus A24 (CV-A24) is a human enterovirus that causes acute flaccid paralysis. However, a Coxsackievirus A24 variant (CV-A24v) is the most common cause of eye infections. The causes of these variable pathogenicity and tissue tropism remain unclear. To elucidate the phylodynamics of CV-A24 and CV-A24v, we analyzed a dataset of 66 strains using Bayesian phylodynamic approach, along with detailed sequence variation and epistatic analyses. Six CV-A24 strains available in GenBank and 60 CV-A24v strains, including 11 Taiwanese strains, were included in this study. The results revealed striking differences between CV-A24 and CV-A24v exhibiting long terminal branches in the phylogenetic tree, respectively. CV-A24v presented distinct ladder-like clustering, indicating immune escape mechanisms. Notably, 10 genetic recombination events in the 3D regions were identified. Furthermore, 11 missense mutation signatures were detected to differentiate CV-A24 and CV-A24v; among these mutations, the F810Y substitution may significantly affect the secondary structure of the GH loop of VP1 and subsequently affect the epitopes of the capsid proteins. In conclusion, this study provides critical insights into the evolutionary dynamics and epidemiological characteristics of CV-A24 and CV-A24v, and highlights the differences in viral evolution and tissue tropism.

## 1. Introduction

Coxsackievirus A24 (CV-A24) is a nonenveloped, positive-sense, single-stranded RNA virus classified under the enterovirus (EV) genotype C in the *Picornaviridae* family [1]. While most EV infections induce limited symptoms, CV-A24 is associated with acute flaccid paralysis, resulting in substantial muscular weakness and acute neurological disorders [2,3]. Certain substitutions in the CV-A24 genome have been reported to enhance virulence or alter cellular tropism, thus shifting systemic infection to restricted tissue [4,5]. The Coxsackievirus A24 variant (CV-A24v) is an antigenic mutant of the CV-A24 strain and is known for its potential to cause outbreaks. Since the 1970s, CV-A24v has been responsible for outbreaks of acute hemorrhagic conjunctivitis (AHC), which is marked by severe eye pain and subconjunctival bleeding [6,7]. In addition, CV-A24v has occasionally been isolated during AFP surveillance [7,8]. CV-A24 and CV-A24v can utilize Intercellular Adhesion Molecule 1 (ICAM-1) as a cellular receptor. However, CV-A24v has also shown enhanced capability to bind to sialic acid and facilitate infection of corneal cells [9]. Despite these findings, the exact pathogenicity and tissue tropism of CV-A24v, as well as its relationship to CV-A24, remain unclear.

Viral phylodynamics and phylogeographics can crucially contribute to the comprehensive knowledge of epidemiological trends and genetic variation in pathogens, as well as their transmission history. These analyses illustrate how interactions between viral genome sequences and molecular functions influence evolution [10]. With evolving viral dispersal patterns, the importance of exploring tree topology characteristics with spatial and temporal components involved in viral succession increases, and it helps deduce the evolutionary history of viruses [11].

Epistasis refers to the phenomenon wherein the effect of one gene on a trait is modified by the expression of one or more other genes, thereby significantly influencing evolutionary dynamics [12]. Genes typically interact with each other in complex patterns, which can substantially impact phenotypes [13]. A thorough understanding of epistasis can elucidate how these interactions affect the expression of specific characteristics, potentially explaining the variable tissue tropisms observed in CV-A24 and CV-A24v.

Mutations and recombination associated with the lack of proofreading by RNA-dependent RNA polymerase (RdRp) led to the emergence of new viral variants [14]. Analysis of the historical spatiotemporal transmission pattern and dynamics of viral populations potentially supports understanding the viral genetic diversity and provides insights into viral succession and circulation trends. Hence, in this study, we aimed to elucidate the relationships between viral evolution, patterns of epidemic transmission, and recombination among CV-A24 and CV-A24v viral strains using integrated Bayesian phylodynamic and epistatic analyses.

## 2. Materials and Methods

### 2.1. Specimen Collection and Ethics Statement

In this study, ethical approval was granted by the Institutional Review Boards (IRB). Enterovirus strains were randomly chosen from a pool of viral stocks isolated from routine eye swab specimens at the KMUH. While investigating the characteristics of the isolated viral strains, we delinked the samples from personal identifiers because no medical history or clinical information was required for the tree topology study.

### 2.2. Viral RNA Extraction, RT-PCR, and Viral Genome Sequencing

Viral strains were propagated using rhabdomyosarcoma (RD) cells and purified. For this purpose, cell debris and large particles were removed from the culture through centrifugation, and the viral aliquot was preserved at −80 °C. We extracted viral RNA, using the QIAamp^®^ Viral RNA mini kit (Qiagen, Chatsworth, CA, USA) following the instructions provided by the manufacturer. Purified RNA was qualitatively assessed using an RNA 6000 Nano Kit and Agilent 2100 Bioanalyzer (Agilent Technologies; Santa Clara, CA, USA) that supports electrophoretic separation of RNA fragments, allowing visualization of the RNA profile and assessment of its integrity and purity. The cDNA library was constructed using Ovation^®^ RNA-Seq System V2 (NuGEN; Redwood, CA, USA) [15], followed by the fragmentation of cDNAs using a Covaris S220 instrument (Covaris; Woburn, MA, USA).

The fragmented cDNAs were ligated to an adapter using the Ovation Ultralow Library System V21-96 (NuGEN; Redwood, CA, USA). The Qubit^®^ 2.0 Fluorometer and High Sensitivity DNA kit (Invitrogen, Thermo Fisher Scientific; Waltham, MA, USA) were used for library control. Product sequencing was conducted on an Illumina MiSeq platform (Illumina; San Diego, CA, USA) using 2 × 300 bp paired-end sequencing. All kits were used according to the instructions provided by the manufacturer. Viral reads were analyzed and de novo assembled using the QIAGEN CLC Genomics Workbench v.9.5 (QIAGEN; Redwood, CA, USA). Eleven viral isolates were identified as CV-A24v using whole-genome sequencing.

### 2.3. Sequence Variation and Phylodynamic Analysis

To reconstruct the demographic and spatiotemporal transmission of CV-A24 and CV-A24v, a comprehensive dataset was compiled using the NCBI (1953 sequences, date: 20231126). Nonaligned strains, which do not consist of the whole genome sequences, were excluded, and representative strains were chosen randomly from those isolated in the same year and geographical area. Eventually, a dataset of 66 strains, including 6 CV-A24 and 60 CV-A24v strains, was generated. CV-A24v strains collected between 1952 and 2017 included 11 Taiwanese strains (Table S1) and 49 strains from 17 other countries (Table S2).

A phylogenetic tree was constructed following the maximum likelihood (ML) method using Molecular Evolutionary Genetics Analysis (MEGA) software (v11) under the GTR+G+I model [16]. To ensure that the tree topology had sufficient temporal signals, a root-to-tip regression analysis was performed using the TempEST program [17,18,19]. We used the Bayesian Evolutionary Analysis Sampling Tree (BEAST) program (v1.10.4) [20] to calculate evolution rates, and the mean of the most recent common ancestor (TMRCA) to elucidate viral demographics and spatiotemporal transmission. The path sampling (PS) and stepping-stone (SS) approaches were used to evaluate the potential best-fit model compositions: a substitution, three clock molecular models, and four tree models (Table S3) [8,18,21]. 

Bayesian analyses were conducted for 45 million states, and sampling was performed once every 45,000 generations until the distributions were stationary. The parameter traces were monitored using the Tracer v.1.7.2 program, with effective sample size values of >200. A maximum clade credibility tree was constructed using Tree-Annotator and visualized using FigTree v.1.4.4 (http://tree.bio.ed.ac.uk/software/figtree/, accessed on 7 August 2024). Discrete phylodynamic analyses of both viruses were performed using Bayesian estimates and annotated using the Spatial Phylogenetic Reconstruction of Evolutionary Dynamics 3 (SpreaD3) software. We explored the viral transmission routes through Google Earth, and significant transition rates were identified using a Bayesian factor (BF) > 3.

### 2.4. Detection of Sequence Variation

The Recombination Detection Program v4.45 (RDP 4) and SimPlot were used to discover potential recombination events and identify recombination breakpoints in the sequences [22,23]. Subsequently, the sequences were analyzed using MEGA 11 and phylogenetic trees were constructed. The neighbor-joining (NJ) method was used to generate phylogenetic trees based on the detected breakpoints, which depicted the evolutionary relationships among recombinant sequences, major parental sequences, minor parental sequences, and reference sequences. MEGA11 and DataMonkey websites were used to analyze sequence comparisons and epistatic interactions, respectively. The Spidermonkey/Bayesian graphical model (BGM) was used to detect sites with epistatic interactions, and PP > 0.5 was considered statistically significant.

### 2.5. GenBank Accession Numbers

The genome sequences of 11 Taiwan CV-A24v strains analyzed in this study were deposited in the GenBank database under accession numbers PP261373–PP261374 and PP265991–PP265999 (https://www.ncbi.nlm.nih.gov/genbank/, accessed on 3 February 2024).

## 3. Results

### 3.1. Phylodynamic Trees Constructed from the Genome

**Figure 1:**
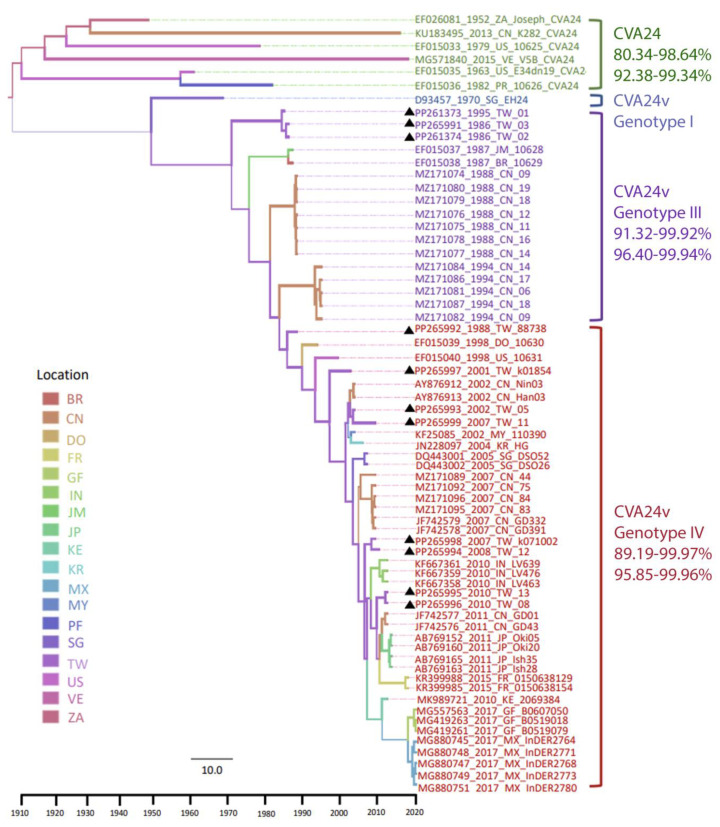
Maximum clade credibility (MCC) tree for 66 worldwide strains of Coxsackievirus A24 and its variant. This tree was constructed using genome sequences, including six CV-A24 strains and sixty strains of CV-A24v, through the Bayesian method with the BEAST program. Branch thickness indicates state probability, and color coding represents the most probable locations. The tree illustrates the proportional relationship between branch length and time, with the dashed line at the bottom indicating the timescale bar. The color codes for ten analyzed locations are shown on the left of the phylogeny. On the right, strain names are denoted by accession number–year–country abbreviation–strain name, with 11 Taiwan genome sequences marked with triangles. Each CV-A24, CV-A24v, and genotype is indicated on the right. Different text colors represent various genotypes: green for CV-A24, blue for genotype I, purple for genotype III, and red for genotype IV. The nucleotide/amino acid similarity (%) for each genotype is indicated in the right column. Abbreviations for locations: BR: Brazil; CN: China; DO: Dominica; FR: France; GF: French Guiana; IN: India; JM: Jamaica; JP: Japan; KE: Kenya; KR: Korea; MX: Mexico; MY: Malaysia; PF: Puerto Rico; SG: Singapore; TW: Taiwan; US: the United States; VE: Venezuela; ZA: South Africa.

The Yang96 substitution, the uncorrelated relaxed clock, and Gaussian Markov random field (GMRF) SkyGrid models were considered as the best model compositions to construct the Bayesian Monte Carlo Markov Chain (BMCMC) tree using the genome sequence dataset. The time of TRMCA was 1918 (95% highest probability density (HPD) interval, 1905–1929), and the evolution rate was estimated as 4.29 × 10^−3^ substitutions/site/year (95% HPD interval: [3.29 × 10^−3^, 5.45 × 10^−3^]). The ML and BMCMC-based trees were constructed using four distinct groups: CV-A24, the prototype CV-A24v, genotype (G) III (isolated between 1985 and 1994), and GIV (isolated after 2000) [24,25,26] (Figure 1). 

Moreover, the phylogeny revealed that these four distinct groups were isolated based on their timespans, rather than their isolation locations. After the first isolation of CV-A24 in South Africa (1952), it reemerged in the United States (1963, 1979), Puerto Rico (1982), China (2013), and Venezuela (2015). GIII was reported in strains isolated in Taiwan (1985–1986), Jamaica (1987), Brazil (1987), and China (1988, 1994). Most GIV strains were identified after the 2000s, which included isolates from Taiwan (1988, 2001–2001, 2004–2005, 2007–2008, 2010), the Dominican Republic (1993), the United States (1998), Malaysia (2002), China (2002, 2007, 2010), South Korea (2004), Singapore (2005), India and Kenya (2010), Japan (2011), France (2015), Mexico, and French Guiana (2017) (Tables S1 and S2). The root-to-tip regression analysis of only CV-A24v revealed an increase in R square from 0.46 to 0.85. However, the total dataset exhibited an insufficient temporal signal, which potentially contributed to the limited number of CV-A24 strain sequences. However, the results of the ML and BMCMC methods revealed similar topologies; both temporal and regional aggregation characteristics were detectable, and, hence, the selected data were reliable [8] (Figure S1).

The phylogeny exhibited an unbalanced topology with a ladder-like backbone, supported by high values on the major branches. This ladder-like structure indicates strong directional selection, leading to an imbalanced tree. Successful lineages form the backbone, while new outbreaks caused by emerging strains quickly result in short terminal branches due to rapid replacement. Herd immunity perpetuates this cycle by trimming these lineages, leaving short terminal ends and creating a noticeably unbalanced phylogenetic tree. Each CV-A24 strain had a single long terminal branch. Contrastingly, CV-A24v exhibited a unique evolutionary pattern characterized by a ladder-like clustering structure based on time, with shorter terminal branches. These discrepancies reflect complicated evolutionary mechanisms that have potentially influenced the diversity and adaptation of CV-A24 and CV-A24v.

### 3.2. Viral Demographic History and Geographic Transmission

**Figure 2:**
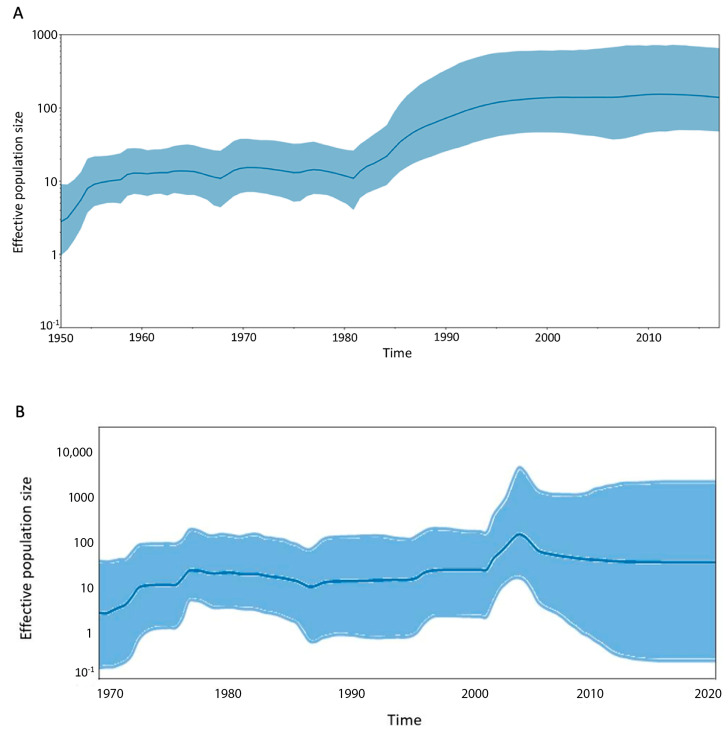
Demographic history of Coxsackievirus A24 and its variant. Two datasets were analyzed: (**A**) 66 strains, including both CV-A24 and CV−A24v strains, and (**B**) 60 CV-A24v strains, focusing specifically on the CV-A24v variant. In the resulting graphs, the *X*-axis represents the timescale in years, while the *Y*-axis displays the logarithmic Neτ scale, where Ne denotes the effective population size and τ represents the generation time. The thick solid line indicates the median estimates from the SkyGrid analysis, and the shaded area represents the 95% highest posterior density interval.

To estimate the trajectories of viral effective population size over time, we utilized the GMRF SkyGrid model. This Bayesian nonparametric model is especially effective for visualizing temporal changes in population dynamics using genetic data. In the resulting graph (Figure 2), the *X*-axis represents the time in years, while the *Y*-axis depicts the effective population size. The absolute values on the *Y*-axis illustrate trends rather than providing precise counts. The curve has increased since the introduction of CV-A24 in the 1950s. CV-A24v was first identified in the 1970s, and its population gradually declined; however, the viral population expanded in the 1980s, causing the curve to drop drastically. The curve slightly increased from 1990 onward, reaching its peak in the 2000s for CV-A24v. A plateau was noticed after 2000, indicating stability in the viral population. Moreover, this plateau coincided with a re-emergence period, associated with the rapid spread of the virus to numerous countries worldwide. In the analyses of discrete phylodynamic routes, only two epidemiological linkages were identified with BF > 3: transmission from Taiwan to Kenya and from Jamaica to Brazil. Notably, despite the occurrence of CV-A24 and CV-A24v epidemics in many nations, the transmission patterns appear to be affected by chronology rather than geographic connections.

### 3.3. Recombination and Epistatic Analysis 

**Figure 3:**
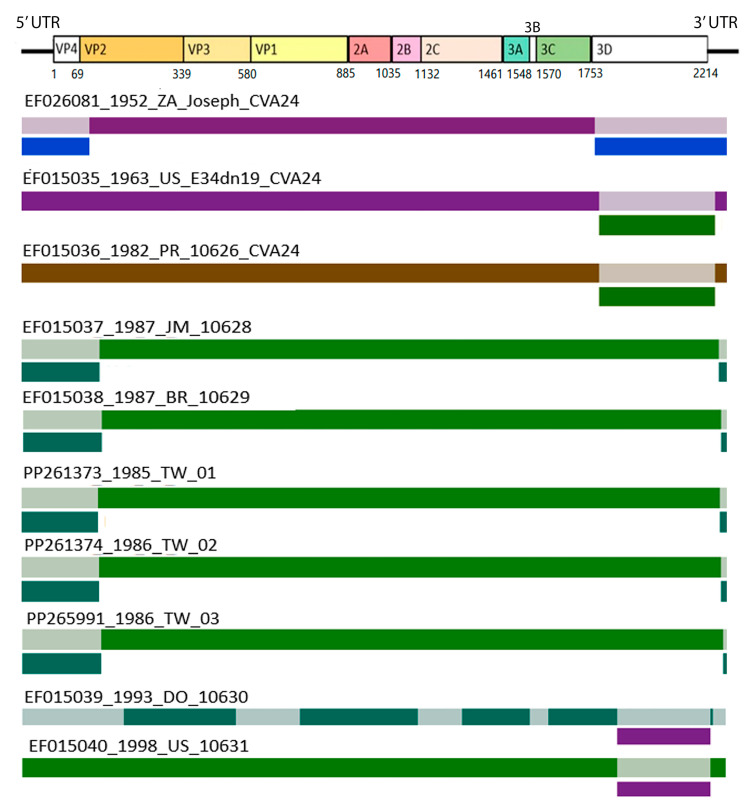
Breakpoints of recombination sequences. The graph above illustrates the coxsackievirus genome, emphasizing the breakpoints of recombination sequences. This genome contains an open reading frame (ORF) flanked by the 5′ and 3′ untranslated regions (UTRs). The ORF is subdivided into structural protein coding regions (VP4, VP2, VP3, and VP1) and nonstructural protein coding regions (2A–2C and 3A–3D). The connected coding sites are indicated beneath their respective coding region cut sites. Additionally, ten strains exhibiting recombination events are aligned below the genome illustration. Each strain is labeled with its name and represented by colored segments, indicating sequence similarity. The colored segments represent nonrecombination regions, while gray segments, with a color bar below, indicate recombination regions. These recombination regions meet the *p*-value threshold of <0.05. These ten schematic diagrams of potential recombinant regions and breakpoint locations were identified by the Recombination Detection Program v3.44 (RDP).

In this study, we identified ten recombination events (Figure 3), mostly in the 5′ untranslated region (UTR), 3D, and 3′UTR. The breakpoints were primarily located in the 3D region, involving the CV-A24 prototype strain (EF026081), the United States and Puerto Rico strains reported in 1963 and 1982 (EF015035 and EF015036), and the Dominican Republic and United States strains reported in 1993 and 1998 (EF015039 and EF015040). Except for EF015039 and EF015040, all recombination events exhibited breakpoints in untranslated regions (UTRs). Moreover, a breakpoint in the VP4 region was detected in the strains EF015037, EF015038, PP261373, PP261374, and PP265991. Bootscan results revealed potential recombination sites, whereas SimPlot findings revealed similarities between the major, minor, and reference sequences. Despite no designated hotspots, recombination sites have emerged in particular areas. The data are depicted in Figure S2 and Table S4.

**Figure 4:**
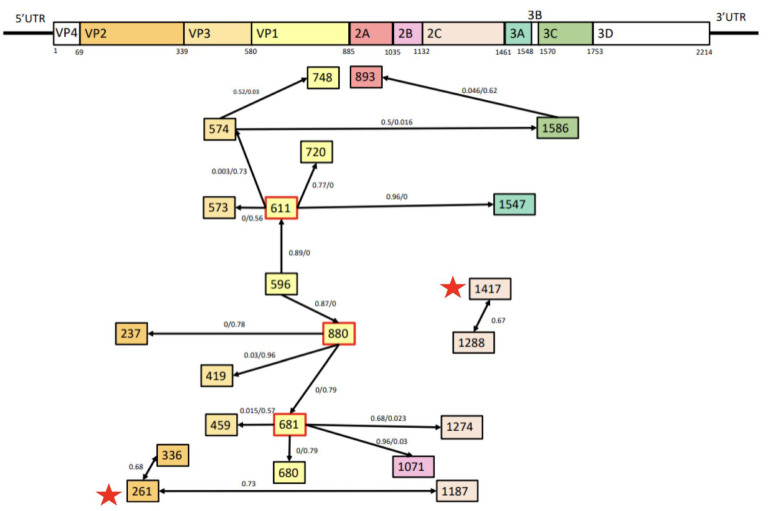
Epistatic interactions between different loci in the whole genome. Each square in the diagram represents the position of a residue involved in at least one interaction; a marginal posterior probability (PP) exceeding the default cut-off of 0.5 was considered. Red squares indicate the positions involved in multiple epistatic interactions. Arrows connecting squares indicate the direction of epistasis. The red pentagram indicates the same mutation sites as found in Figure 5. The associated PP values are presented in the following sequence: PP(→)/PP(↔)/PP(←). The upper numbers are PP between positions. These epistatic interactions have been identified using BGM on the Datamonkey website. The positions marked with stars represent the mutation positions with epistatic interactions.

**Figure 5:**
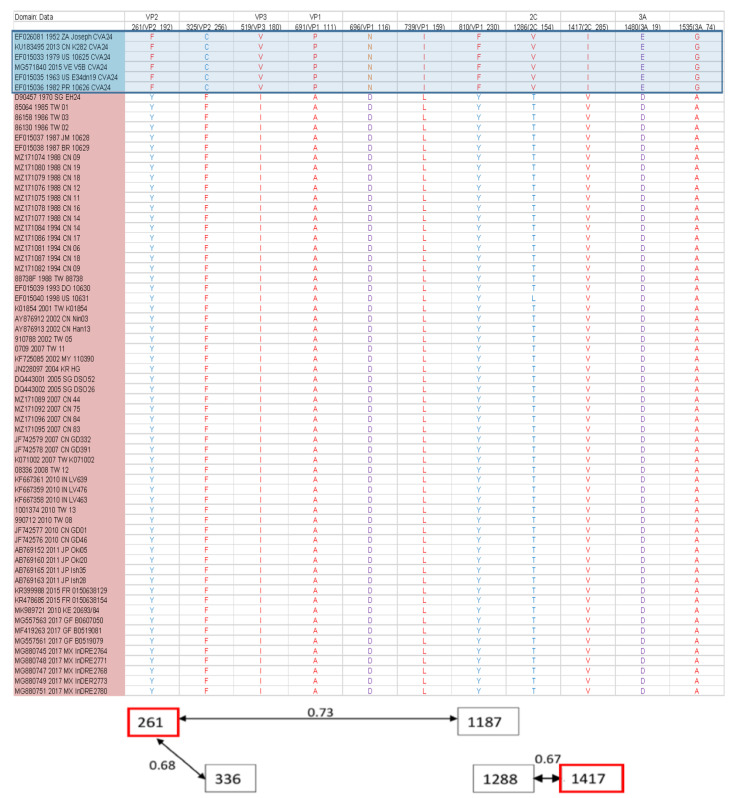
Missense mutation signatures between CV-A24 and its variants. This table lists the common missense mutations between CV-A24 and its variants. The first row indicates the gene region where the mutation occurs. The second row notes the mutation site within the entire sequence (site in the gene region). The upper six rows, shaded in light blue, represent the CV-A24 strains. Amino acids are color-coded based on their properties: blue for hydrophilic, red for hydrophobic, purple for acidic, and orange for basic. Epistatic interactions at the signature mutation sites (highlighted in the red box) are shown at the bottom. A marginal posterior probability (PP) exceeding the default cutoff of 0.5 was considered significant. Arrows connecting squares indicate the direction of epistasis between residues, with PP values presented as PP(→)/PP(?)/PP(←). Residue abbreviations: A, Alanine; C, Cysteine; D, Aspartic acid; E, Glutamic acid; F, Phenylalanine; G, Glycine; I, Isoleucine; L, Leucine; N, Asparagine; P, Proline; T, Threonine; V, Valine; Y, Tyrosine.

The comparative analysis of the amino acid sequences of CV-A24 and CV-A24v revealed that missense mutations occur in various gene regions in each strain. However, a distinct group of eleven common missense mutations differentiates CV-A24 from its variant. These signature mutations are F261Y, C325F, V519I, P691A, N696D, I739L, F810Y, V1286T, I1417V, E1480D, and G1535A (Figure 4). Among these, three hydrophobic amino acids changed to hydrophilic ones (F261Y on VP2, F810Y on VP1, and V1286T on 2C), a hydrophilic amino acid changed to a hydrophobic one (C325 F on VP2), and a basic amino acid was altered to an acidic one (N696D on VP1). The epistatic analysis revealed 22 pairs of interactions (PP > 0.5). Notably, positions 661, 681, and 880 displayed numerous epistatic interactions with more than four other sites, predominantly within the VP1 region (Figure 5). Furthermore, F261Y exhibited epistatic interactions with positions 336 and 1187. I1417V exhibited epistatic interactions with position 1288. Overall, these findings illuminate the genetic variations and interactions between CV-A24 and CV-A24v, offering insights into their molecular features, potential pathogenicity, and evolutionary dynamics.

## 4. Discussion

Although CV-A24 infection causes systemic illness, CV-A24v primarily causes ocular disease [2,3,26]. The causes of these discrepancies remain to be determined. Several studies have attempted to investigate the origin and dynamic evolution of CV-A24v based on the VP1 and 3C regions [5,24,25,27,28]. Furthermore, to understand the relationship between sequence variants and tissue tropism, a previous study analyzed the VP1 sequences of CV-A24v from ocular and gut tissues. This analysis revealed an association of two novel genotypes with AFP and identified positive selection at residue 146T, indicating potential adaptation during the CVA24v pandemic [8]. However, other AFP isolate sequences clustered with AHC isolates, especially after 2000. To further elucidate these findings, we conducted additional phylodynamic analyses of CV-A24 and CV-A24v using genome sequences and identified four distinct groups of Coxsackie A viruses with the BMCMC method [5,24,25]: CV-A24, the prototype CV-A24v, G III, and GIV [24,25,26]. CV-A24 and the prototype CV-A24v represent separate branches. However, while CV-A24 has elongated branches, CV-A24v has shorter exterior branches., indicating that CV-A24v experiences relatively consistent herd selection pressure [29]. The BMCMC-based chronological trend in CV-A24v outbreaks highlighted a dynamic evolution of the virus over time. The ladder-like backbone indicates the successful lineages. New outbreaks are caused by emerging strains that quickly lead to short terminal ends owing to their rapid replacement. The cycle is subsequently perpetuated by herd immunity, which trims these lineages, leaving short-terminal branches, resulting in a phylogenetic tree with a noticeably unbalanced topology [29,30,31]. Despite the availability of CV-A24 sequences in GenBank, the phylogeny provided strong support for the main backbone.

Based on the 3C^pro^ and VP1 region, our previous studies have categorized CVA24v into four genotypes (GI-GIV) [5,24]. Genotype I consists of the prototype strain alone. Genotype II strains were isolated in the 1970s in Asia. However, no existing GII viral genome sequences have been deposited in GenBank, except those based on the 3Cpro sequence. Genotype III strains were isolated from 1980 to 1990 in Asia. These findings imply that topologies produced by VP1-based and genome-based methods are comparable, particularly in clades with high support values. Additionally, the statistics show that GIV has become the main genotype and has been spreading quickly around the world since 2000, whereas GI-GIII has become dormant during the previous few decades.

Viral populations and transmission routes explain the causes and patterns of outbreaks [32]. Previous studies reported four separate stages of CV-A24v outbreaks, which included the first isolation in the 1970s, substantial increases in the 1980s, stabilized prevalence since the 1990s, and a resurgence in the 2000s [31]. In this study, the introduction of CV-A24 in the 1950s was followed by an initial increase and a decline after the identification of CV-A24v. A surge in CV-A24v outbreaks across Northeast Asia and the Western Hemisphere was reported in the 1980s [33,34,35,36]. Moderate outbreaks in the 1990s reflected a silent period [25,31]. A peak during the mid-2000s indicated a re-emergence of the variant. These phases highlight the adaptive transmission dynamics of viruses over time. [24,31]. The demographic history depicted in Figure 3 reveals significant trends in the global population dynamics of CV-A24v. Phylodynamic analysis identified two epidemiological linkage routes with a Bayesian factor (BF) > 3, which reflects transmission from Taiwan to Kenya and from Jamaica to Brazil. Interestingly, despite outbreaks in multiple countries, no geographical influence on the transmission trends was evident.

Enterovirus C was reported to exhibit a remarkably high recombination rate [37], particularly at the edges of the structural P1 region or within 5′UTR, P2, or P3 regions [38,39,40,41]. While recombination can occur throughout the genome, not all recombinants are viable. Recombinants with breakpoints in certain regions are potentially preferred [39,42]. Our study demonstrated ten recombination events that shed light on the genomic diversity of CV-A24 and CV-A24v. Breakpoints were predominantly located in specific regions, notably the 5′UTR, 3D, and 3′UTR regions, consistent with previous reports [41]. In RNA viruses, recombination usually happens via a copy-choice mechanism. In the process of negative-strand synthesis, RNA-dependent RNA polymerase (RdRp), which is encoded by the 3D gene, switches template strands. This step is crucial for sub-genomic transcription and full-genome replication [43]. Hotspots for template switching between distinct strands are also likely to be found in regions that are hotspots for RdRp jumping within the same strand. The poly(A) tail and 3′UTR are crucial for translation and replication in positive-strand RNA viruses. When the poly(A) tail lands on the 5′UTR of the viral Internal Ribosome Entry Site (IRES), it interacts with host proteins and, in conjunction with the 3′UTR, enhances host ribosome function [44]. This might explain the frequent recombination in 5′UTR, 3D, and 3′UTR regions. 

Some recombinant sequences are associated with ambiguous information on the isolation year preceding that of their parental sequences. Furthermore, recombination between CV-A24 and CV-A24v is detected in recombinant strains identified in the United States in 1963 and Puerto Rico in 1982, which implies genetic exchange between the CV-A24 and CV-A24v lineages. These circumstances are potentially attributable to dendrogram closeness or a lack of relevant nucleotides in the parent sequences [22]. The CV-A24 prototype strain (EF026081) exhibited a closer relation to the ancestral and parental sequences than the CV-A24v prototype strain (D90457). The recombination events likely occurred between CV-A24 and CV-A24v [45]. Additionally, the CV-A24 strains isolated in the United States (EF015035) and Puerto Rico (EF015036), and CV-A24v strains isolated in Taiwan (PP261373, PP261378, PP265991), Jamaica (EF015037), and Brazil (EF015038), are considered potential major or minor parents. 

Amino acid changes may affect the secondary structure and epitopes of capsid proteins [31]. The viral protein VP1 has a conical β-barrel structure comprising eight antiparallel β-strands and includes a hydrophobic pocket in its canyon, which is a key host receptor attachment site. Variations in VP1 often occur in the loops connecting β-strands, notably the BC (residues 97-105) and the GH loops (residues 193-231), which are crucial for immunogenic neutralization and serve as preferred receptor-binding sites on the exterior surface of the virus capsid [46,47,48]. Previous reports demonstrate that the F810Y substitution (residue 230 of VP1) enhances the receptor-binding capacity of the protein, inducing the adaptation of the CV-A24v strain to ocular tropism [9]. These findings highlight the impact of specific amino acid changes in CV-A24v’s biological properties and tropism.

Among the 22 pairs of epistatic interactions, we identified 3 multiple interactions, each involving more than 4 sites; these included interactions at positions 611, 681, and 880, within VP1, crucial for viral binding to cellular receptors [49,50,51]. Position 611 (residue 31 of VP1) is correlated with pathogenicity and enhanced thermostability and immunogenicity [46,51,52]. Position 681 (residue 101 of VP1) is located in the BC loop, which is a critical region for neutralization. Genomic variations, such as recombination, mutation, and interactions, particularly in VP1, affect the pathogenicity and transmission of the virus and potentially shift infection patterns from systemic to ocular tropism.

## 5. Conclusions

This study comprehensively analyzed the genomic and epidemiological characteristics of CV-A24 and its variant, CV-A24v, and provided insights into their evolution, transmission patterns, and potential pathogenic determinants. CV-A24 exhibits distinct, longer terminal branches than CV-A24v, which exhibits a ladder-like structure, suggesting limited tissue tropism. Bayesian phylodynamic analyses estimated the TMRCA of CV-A24 and CV-A24v, which revealed their evolutionary histories. Variations in population over time indicate a greater influence of temporal factors on transmission than that of geographical factories. Ten recombination events in the 5′UTR, 3D, and 3′UTR regions contributed to genetic diversity in the analyzed viral variants. The F810Y mutation and epistatic interactions in the VP1 region significantly affected pathogenicity and transmission. Integrated outcomes of Bayesian phylodynamic and sequence variation analyses contribute to the knowledge of evolutionary relationships, transmission dynamics, and genomic variations in CV-A24 and CV-A24v; moreover, their genetic and clinical differences were elucidated. These findings link recombination, mutations, and epistatic interactions to the shifts in infection patterns from systemic to ocular tropism.

## Data Availability

The data supporting the findings of this study are available in the Methods and/or Supplementary Material of this article.

## Supplementary Materials

The following supporting information can be downloaded at: https://www.mdpi.com/article/10.3390/v16081267/s1, Table S1. The Taiwan strains isolated in this study; Table S2. Reference strains; Table S3. Best-fit model compositions; Table S4. The breakpoint positions. Figure S1. The maximum likelihood tree with R^2^ coefficient of determination; Figure S2. Results of RDP and Simplot software.
