# Supplementary material for: Phylodynamic and Epistatic Analysis of Coxsackievirus A24 and Its Variant
Source: Viruses. 2024 Aug 8;16(8):1267. doi: 10.3390/v16081267 (PMC11359322; doi:10.3390/v16081267)
Supplement: Supplementary file 1 [file viruses-16-01267-s001.zip › viruses-3114632-supplementary.pdf]

Table S1: The Taiwan strains isolated in this study

Accession number	strains	Year
PP261373	TW01	1985
PP261374	TW02	1986
PP265991	TW03	1986
PP265992	TW88738F	1988
PP265993	TW05	2002
PP265994	TW12	2008
PP265995	TW13	2010
PP265996	TW08	2010
PP265997	K01854	2001
PP265998	K071002	2007
PP265999	TW11	2007

Table S2:

Sequence information of the 55 reference strains of CVA24 and CVA24v

Genbank Number	Year	Location
EF026081	1952	South Africa
EF015033	1979	United States
EF015035	1963	United States
EF015036	1982	Puerto Rico
KU183495	2013	Venezuela
MG571840	2015	China
D90457	1970	Singapore
EF015037	1987	Jamaica
EF015038	1987	Brazil
MZ171074	1988	China
MZ171075	1988	China
MZ171076	1988	China
MZ171077	1988	China
MZ171078	1988	China
MZ171079	1988	China
MZ171080	1988	China
MZ171081	1994	China
MZ171082	1994	China
MZ171084	1994	China
MZ171086	1994	China
MZ171087	1994	China
EF015039	1993	Dominican Republic
EF015040	1998	the United States
AY876912	2002	Ningbo, China
AY876913	2002	Hangzhou, China
KF725085	2002	Malaysia
JN228097	2004	South Korea
DQ443001	2005	Singapore
DQ443002	2005	Singapore
MZ171089	2007	China
Genbank Number	Year	Location
MZ171092	2007	China
MZ171095	2007	China
MZ171096	2007	China
JF742578	2007	Guangdong, China
JF742579	2007	Guangdong, China

JF742576	2010	Guangdong, China
JF742577	2010	Guangdong, China
MK989721	2010	Kenya
KF667358	2010	India
KF667359	2010	India
KF667361	2010	India
AB769152	2011	Okinawa, Japan
AB769160	2011	Okinawa, Japan
AB769163	2011	Ishigaki, Japan
AB769165	2011	Ishigaki, Japan
KR399988	2015	France
KR478685	2015	France
MF419263	2017	FRENCH GUIANA
MG557561	2017	FRENCH GUIANA
MG557563	2017	FRENCH GUIANA
MG880745	2017	Mexico
MG880747	2017	Mexico
MG880748	2017	Mexico
MG880749	2017	Mexico
MG880751	2017	Mexico

Table S3. Best-fit model compositions

PS: Tree model/ Clock model	STR	URCG	URClog
CON	-56189	-55602	-55604
BSK	-56163	-55597	-55636
GMRF	-56183	-55581	-55646
EXPO	-56170	-55595	-55599

SS: Tree model/ Clock model	STR	URCG	URClog
CON	-56189	-55602	-55603
BSK	-56163	-55595	-55634
GMRF	-56183	-55580	-55644
EXPO	-56170	Text -55595	-55598

Abbreviations

PS: path sampling

SS: stepping-stone sampling

STR: strict clock

URCG: Uncorrelated clock

URClog: Random local clock

CON: Constant Size

BSK: Bayesian Skyline

GMRF: GMRF Bayesian Skyride

EXPO: Exponential Growth

Table S4 The breakpoint positions

Breakpoint Positions							
In Alignment		In Recombinant Sequence					Detection Methods
Begin	End	Begin	End	Recombinant Sequence(s)	Minor Parental Sequence(s)	Major Parental Sequence(s)	(P<0.05)
718	6024	708	6024	^EF026081_1952_ZA_Joseph_CVA24	D90457_1970_SG_EH24	Unknown (MZ171075_1988_CN_11)	Maxchi, Chimaera, 3Seq
						Unknown(MZ171076_1988_CN_12)	
						Unknown(MZ171078_1988_CN_16)	
						Unknown(MZ171077_1988_CN_14)	
						Unknown(MZ171079_1988_CN_18)	
						Unknown(MZ171074_1988_CN_09)	
						Unknown(MZ171080_1988_CN_19)	
						Unknown(PP265992_1988_TW_88738)	
						Unknown(MZ171081_1994_CN_06)	
						Unknown(MZ171087_1994_CN_18)	
						Unknown(MZ171086_1994_CN_17)	
						Unknown(MZ171082_1994_CN_09)	
						Unknown(MZ171084_1994_CN_14)	
7282*	6069	7275*	6066	^EF015035_1963_US_E34dn19_CVA24	PP265999_2007_TW_11	Unknown (PP261374_1986_TW_02)	Maxchi, Chimaera
				EF015036_1982_PR_10626_CVA24	AY876912_2002_CN_Nin03	Unknown(PP261373_1985_TW_01)	
					AY876913_2002_CN_Han13	Unknown(PP265991_1986_TW_03)	
					PP265993_2002_TW_05	Unknown(MZ171076_1988_CN_12)	
					KF725085_2002_MY_110390	Unknown(MZ171078_1988_CN_16)	
					JN228097_2004_KR_HG	Unknown(MZ171077_1988_CN_14)	
					DQ443002_2005_SG_DSO26	Unknown(MZ171075_1988_CN_11)	
					DQ443001_2005_SG_DSO52	Unknown(MZ171079_1988_CN_18)	
					PP265994_2008_TW_12	Unknown(MZ171074_1988_CN_09)	
					PP265998_2007_TW_K071002	Unknown(MZ171080_1988_CN_19)	
					KF667361_2010_IN_LV639		
					KF667358_2010_IN_LV463		
					KF667359_2010_IN_LV476		
					MG880747_2017_MX_InDRE2768		

837	7292	824	7295	EF015038_1987_BR_10629	Unknown (EF015039_1993_DO_10630)	MZ171075_1988_CN_11	RDP, Boostcan
				EF015037_1987_JM_10628	Unknown(EF015040_1998_US_10631)	MZ171076_1988_CN_12	Maxchi, Chimaera, 3Seq
						MZ171078_1988_CN_16	
						MZ171077_1988_CN_14	
						MZ171079_1988_CN_18	
						MZ171074_1988_CN_09	
						MZ171080_1988_CN_19	
						PP265992_1988_TW_88738	
808	7378	800	7376	PP261374_1986_TW_02	Unknown (PP265999_2007_TW_11)	MZ171075_1988_CN_11	RDP, Boostcan, Maxchi
				PP261373_1985_TW_01	Unknown(AY876912_2002_CN_Nin03)	MZ171076_1988_CN_12	Chimaera, SiScan, 3Seq
				PP265991_1986_TW_03	Unknown(AY876913_2002_CN_Han13)	MZ171078_1988_CN_16	
					Unknown(PP265993_2002_TW_05)	MZ171077_1988_CN_14	
					Unknown(KF725085_2002_MY_110390)	MZ171079_1988_CN_18	
					Unknown(JN228097_2004_KR_HG)	MZ171074_1988_CN_09	
					Unknown(DQ443002_2005_SG_DSO26)	MZ171080_1988_CN_19	
					Unknown(DQ443001_2005_SG_DSO52)	PP265992_1988_TW_88738	
					Unknown(MZ171096_2007_CN_84)	MZ171082_1994_CN_09	
					Unknown(MZ171095_2007_CN_83)		
					Unknown(MZ171092_2007_CN_75)		
					Unknown(JF742578_2007_CN_GD391)		
					Unknown(JF742579_2007_CN_GD332)		
					Unknown(PP265994_2008_TW_12)		
					Unknown(PP265998_2007_TW_K071002)		
					Unknown(MK989721_2010_KE_20693/84)		
					Unknown(KF667361_2010_IN_LV639)		
					Unknown(KF667358_2010_IN_LV463)		
					Unknown(KF667359_2010_IN_LV476)		
					Unknown(AB769160_2011_JP_Oki20)		
					Unknown(AB769152_2011_JP_Oki05)		
					Unknown(AB769163_2011_JP_Ish28)		
					Unknown(AB769165_2011_JP_Ish35)		
					Unknown(JF742576_2010_CN_GD46)		
					Unknown(JF742577_2010_CN_GD01)		
					Unknown(PP265996_2010_TW_08)		
					Unknown(PP265995_2010_TW_13)		
					Unknown(KR399988_2015_FR_0150638129)		
					Unknown(MG557563_2017_GF_B0607050)		
					Unknown(MF419263_2017_GF_B0519081)		
					Unknown(MG557561_2017_GF_B0519079)		
					Unknown(MG880751_2017_MX_InDRE2780)		
					Unknown(MG880749_2017_MX_InDER2773)		
					Unknown(MG880747_2017_MX_InDRE2768)		
					Unknown(MG880745_2017_MX_InDRE2764)		
					Unknown(MG880748_2017_MX_InDRE2771)		
7246*	6247	7236*	6247	^EF015039_1993_DO_10630	EF015035_1963_US_E34dn19_CVA24	EF015037_1987_JM_10628	RDP, Boostcan
				EF015040_1998_US_10631	EF015036_1982_PR_10626_CVA24	EF015038_1987_BR_10629	Maxchi, Chimaera, 3Seq

Table Key:

~ = It is possible that this apparent recombination signal could have been caused by an evolutionary process other than recombination.

* = The actual breakpoint position is undetermined (it was most likely overprinted by a subsequent recombination event).

^ = The recombinant sequence may have been misidentified (one of the identified parents might be the recombinant)

Minor Parent = Parent contributing the smaller fraction of sequence.

Major Parent = Parent contributing the larger fraction of sequence.

Unknown = Only one parent and a recombinant need be in the alignment for a recombination event to be detectable.

NS = No significant P-value was recorded for this recombination event using this method.

Figure S1. The maximum likelihood tree with R^2 coefficient of determination

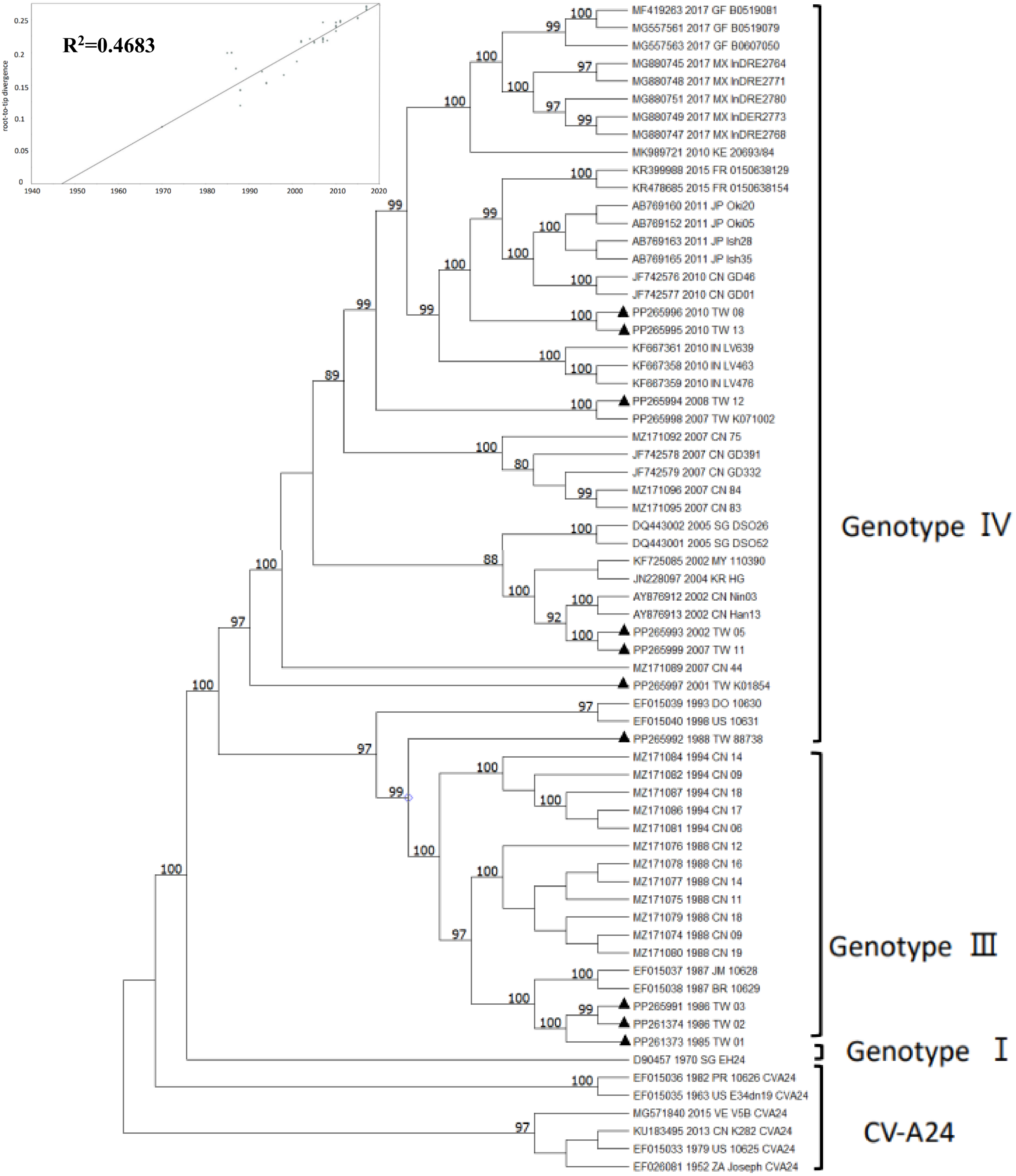

Figure S1(a). Root-to-tip regression analysis estimated with a maximum likelihood (ML) tree. R^2, coefficient of determination. ML tree was constructed from the genome GenBank reference strains and all Taiwan CV-A24v strains identified in this study marked with a triangle were given as accession number-year isolated-country abbreviation-strain name.

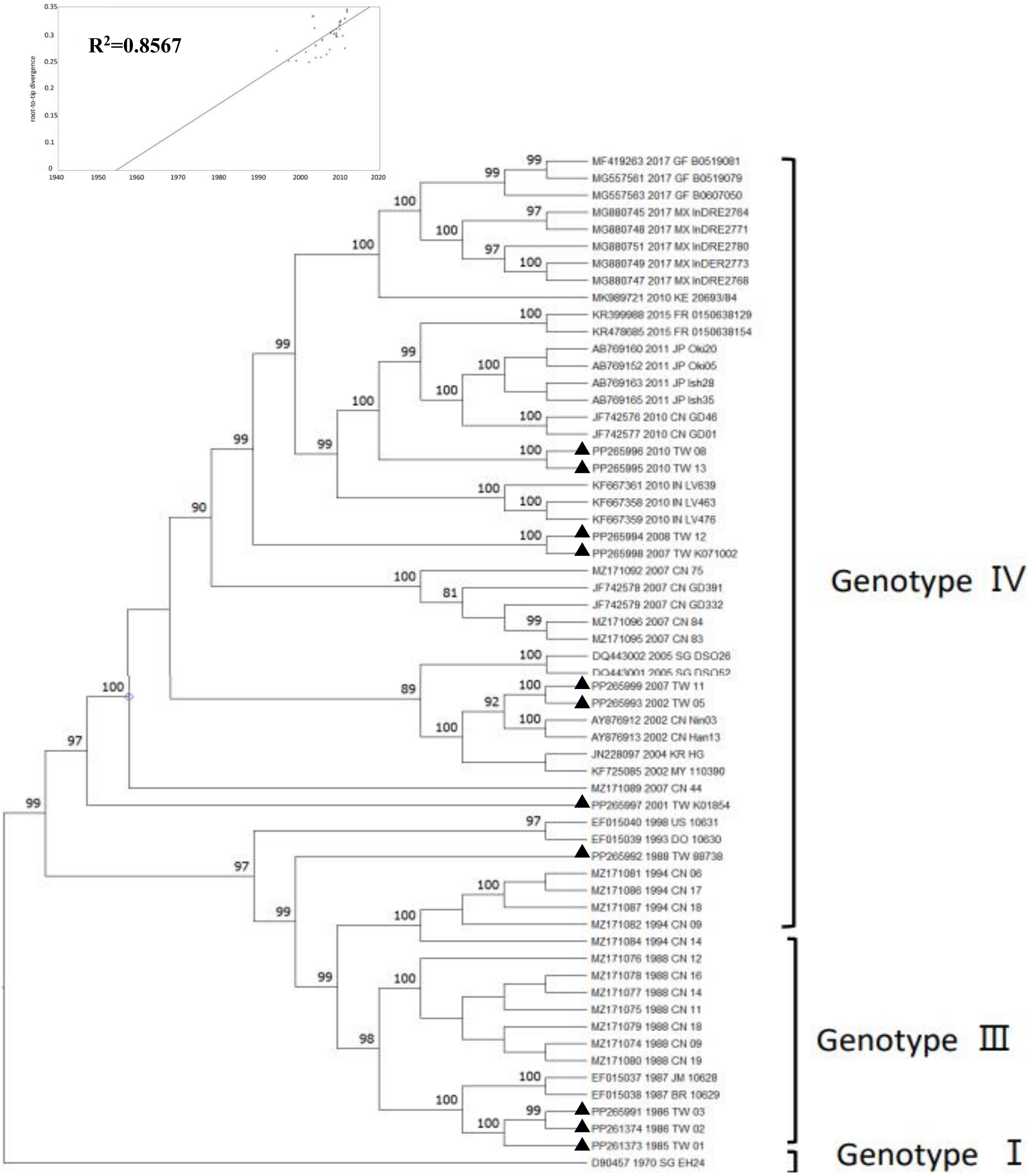

R²=0.8567
root-to-tip divergence
0.35
0.3
0.25
0.2
0.15
0.1
0
1940
1950
1960
1970
1980
1990
2000
2010
2020
MF419263 2017 GF B0519081
MG557561 2017 GF B0519079
MG557563 2017 GF B0607050
MG880745 2017 MX InDRE2764
MG880748 2017 MX InDRE2771
MG880751 2017 MX InDRE2780
MG880749 2017 MX InDER2773
MG880747 2017 MX InDRE2768
MK989721 2010 KE 20693/84
KR399988 2015 FR 0150638129
KR478685 2015 FR 0150638154
AB769160 2011 JP Oki20
AB769152 2011 JP Oki05
AB769163 2011 JP Ish28
AB769165 2011 JP Ish35
JF742576 2010 CN GD46
JF742577 2010 CN GD01
PP265996 2010 TW 08
PP265995 2010 TW 13
KF667361 2010 IN LV639
KF667358 2010 IN LV463
KF667359 2010 IN LV476
PP265994 2008 TW 12
PP265998 2007 TW K071002
MZ171092 2007 CN 75
JF742578 2007 CN GD391
JF742579 2007 CN GD332
MZ171096 2007 CN 84
MZ171095 2007 CN 83
DQ443002 2005 SG DSO26
DQ443001 2005 SG DSO52
PP265999 2007 TW 11
PP265993 2002 TW 05
AY876912 2002 CN Nin03
AY876913 2002 CN Han13
JN228097 2004 KR HG
KF725085 2002 MY 110390
MZ171089 2007 CN 44
PP265997 2001 TW K01854
EF015040 1998 US 10631
EF015039 1993 DO 10630
PP265992 1988 TW 88738
MZ171081 1994 CN 06
MZ171086 1994 CN 17
MZ171087 1994 CN 18
MZ171082 1994 CN 09
MZ171084 1994 CN 14
MZ171076 1988 CN 12
MZ171078 1988 CN 16
MZ171077 1988 CN 14
MZ171075 1988 CN 11
MZ171079 1988 CN 18
MZ171074 1988 CN 09
MZ171080 1988 CN 19
EF015037 1987 JM 10628
EF015038 1987 BR 10629
PP265991 1986 TW 03
PP261374 1986 TW 02
PP261373 1985 TW 01
D90457 1970 SG EH24
Genotype Ⅳ
Genotype Ⅲ
Genotype Ⅰ

Figure S1(b). Root-to-tip regression analysis estimated with a maximum likelihood (ML) tree. R^2, coefficient of determination. The ML tree was constructed from the genome GenBank reference strains without CV-A24 strains and all Taiwan CV-A24v strains identified in this study marked with a triangle were given as accession number-year isolated-country abbreviation-strain name.

Figure S2.

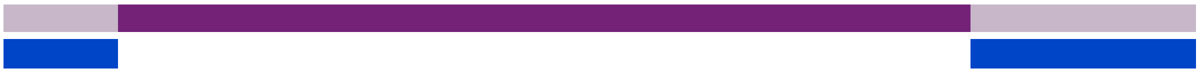

(A)

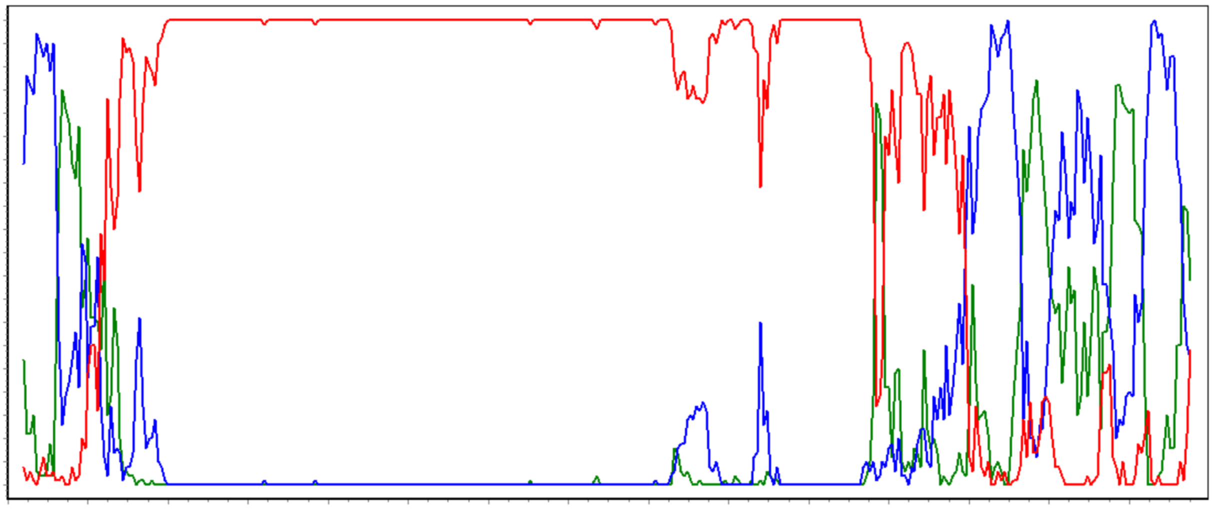

(B)

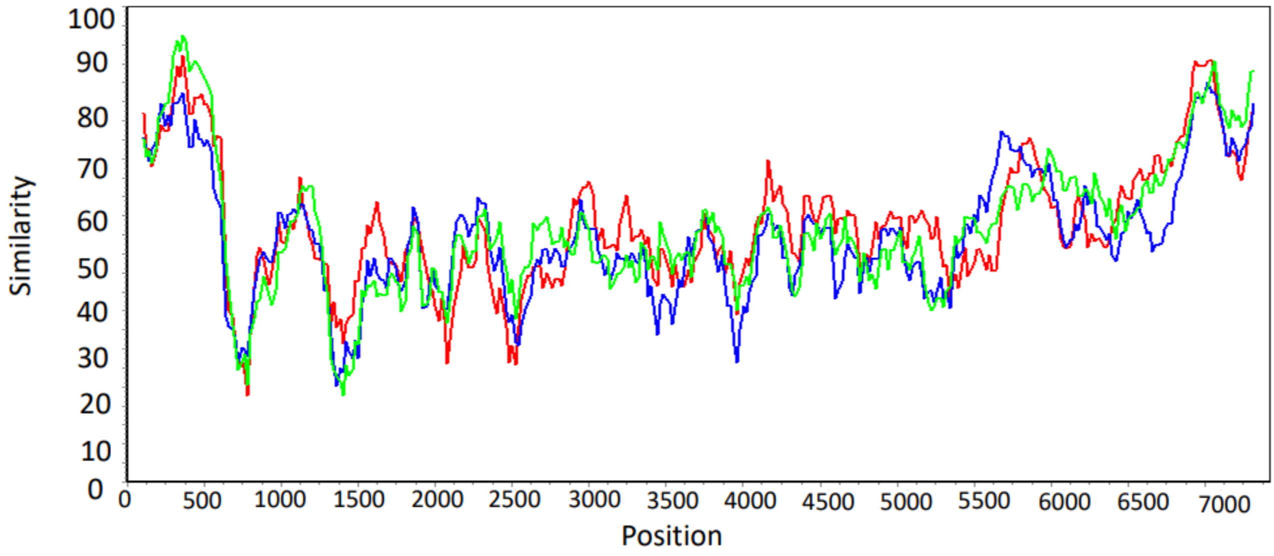
100
90
80
70
60
50
40
30
20
10
0
Similarity
0
500
1000
1500
2000
2500
3000
3500
4000
4500
5000
5500
6000
6500
7000
Position

(C) NJ tree based on recombination fragments (1-718, 6034-7422 nt)

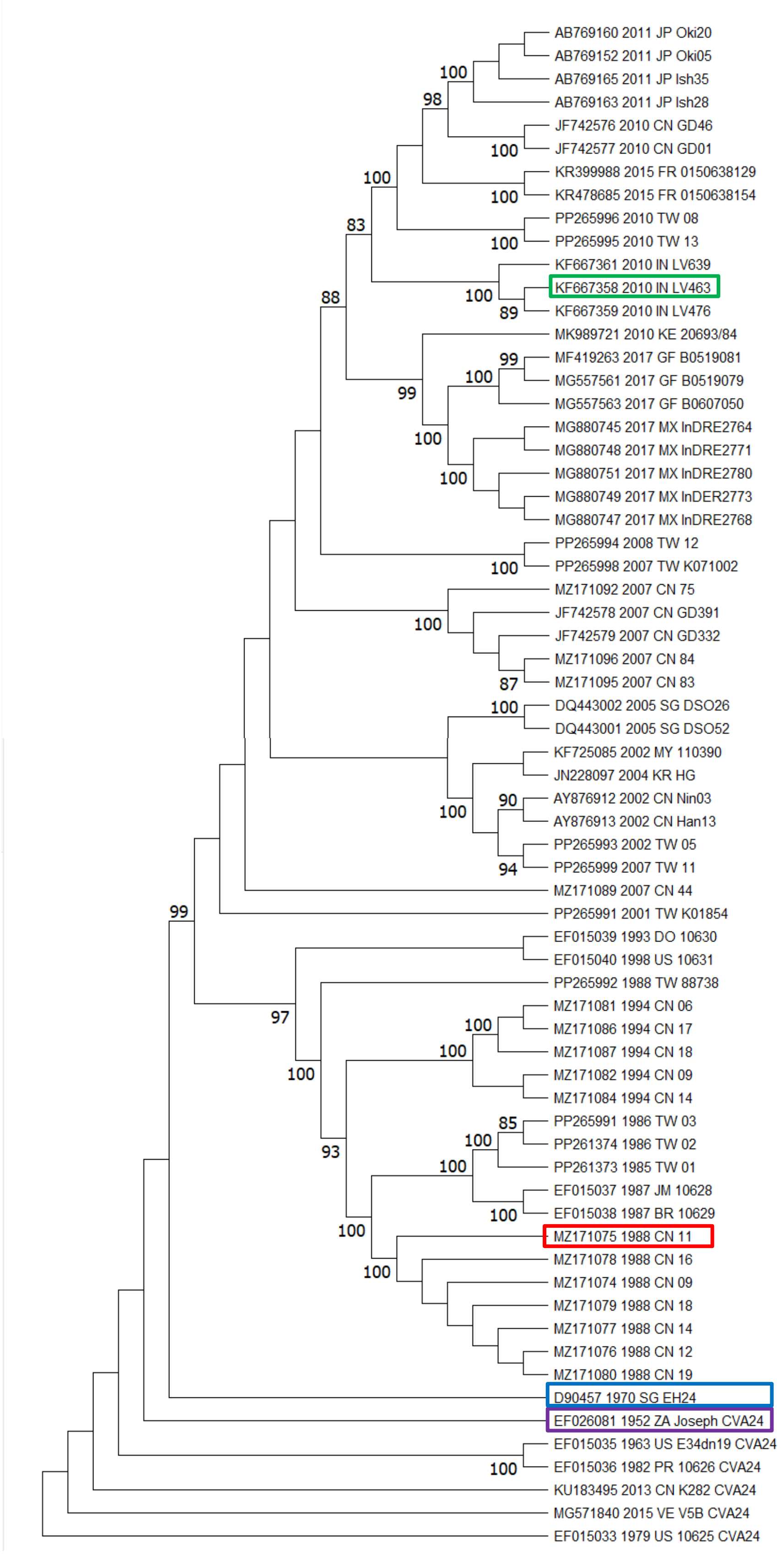

(D) NJ tree based on non-recombination fragments (619-6033 nt)

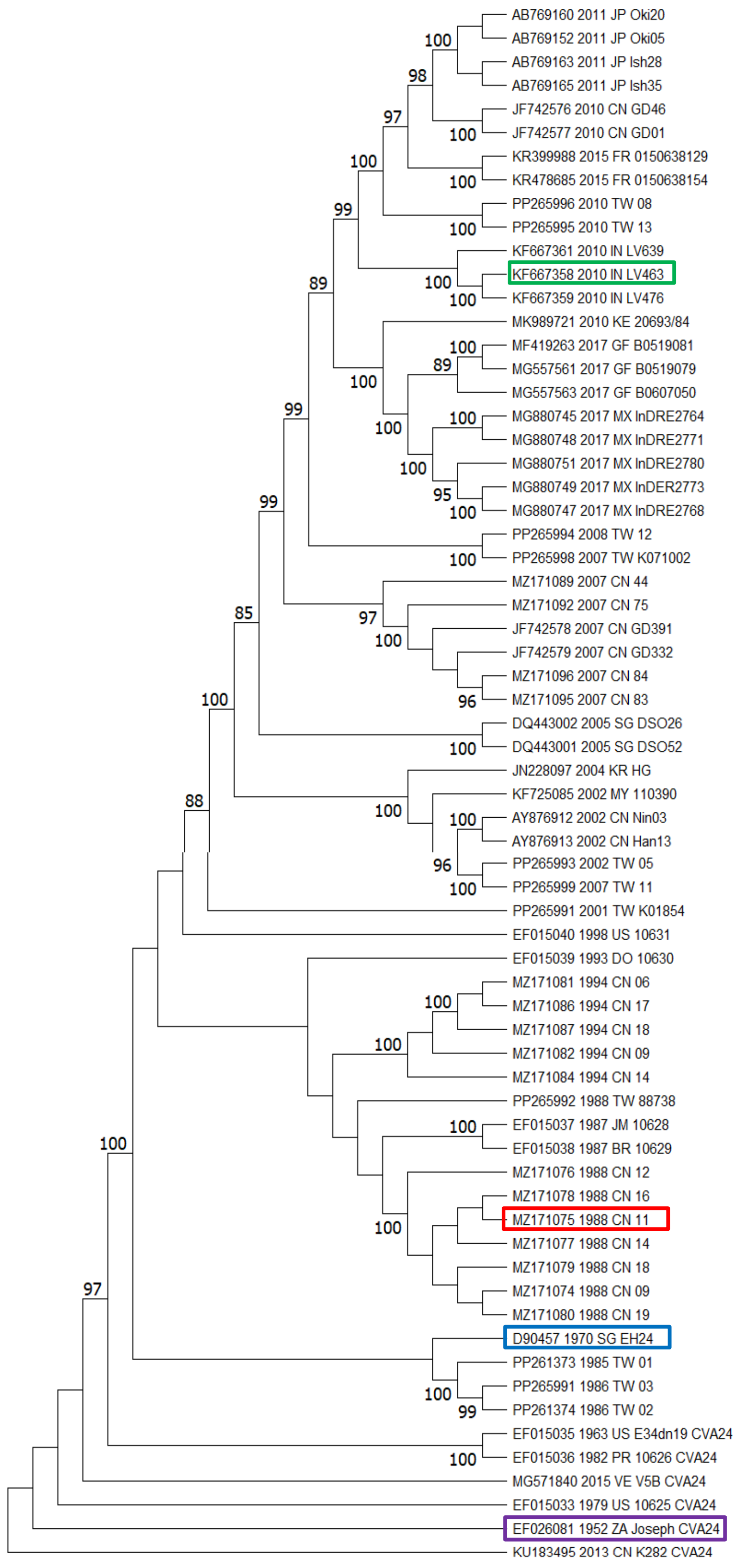

Figure S1(A) Result of Bootscan Figure S1 (B)Result of SimPlot. Color red, blue, and green represent major parental sequence(s), minor parental sequence(s), and reference parental sequence(s). Figure S1(C) NJ tree based on recombination fragments (1-618, 6034-7422 nt) Figure S1 (D) NJ tree based on non-recombination fragments (619-6033 nt). Color red, blue, green, and purple represent major parental sequence(s), minor parental sequence(s), reference parental sequence(s), and recombinant sequence.

CVA24 (EF015035) (EF015036)

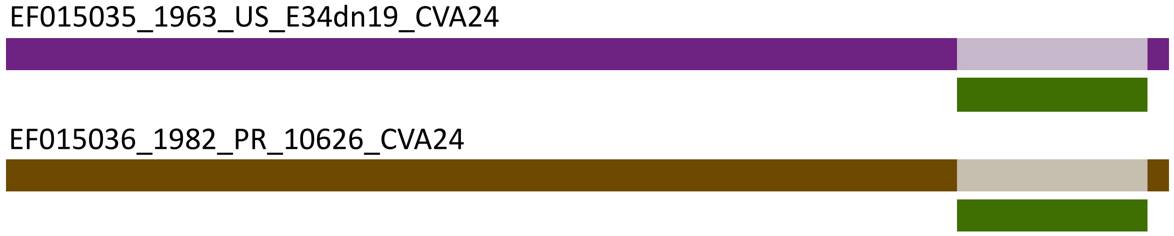

(E) Result of Bootscan

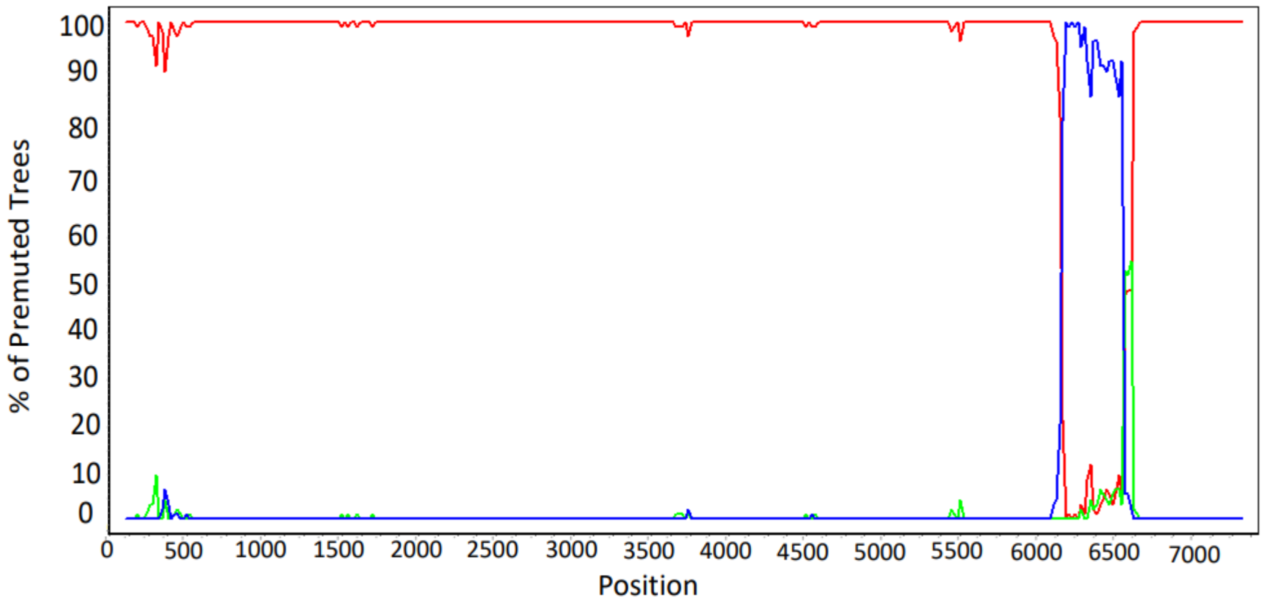

(F) Result of SimPlot

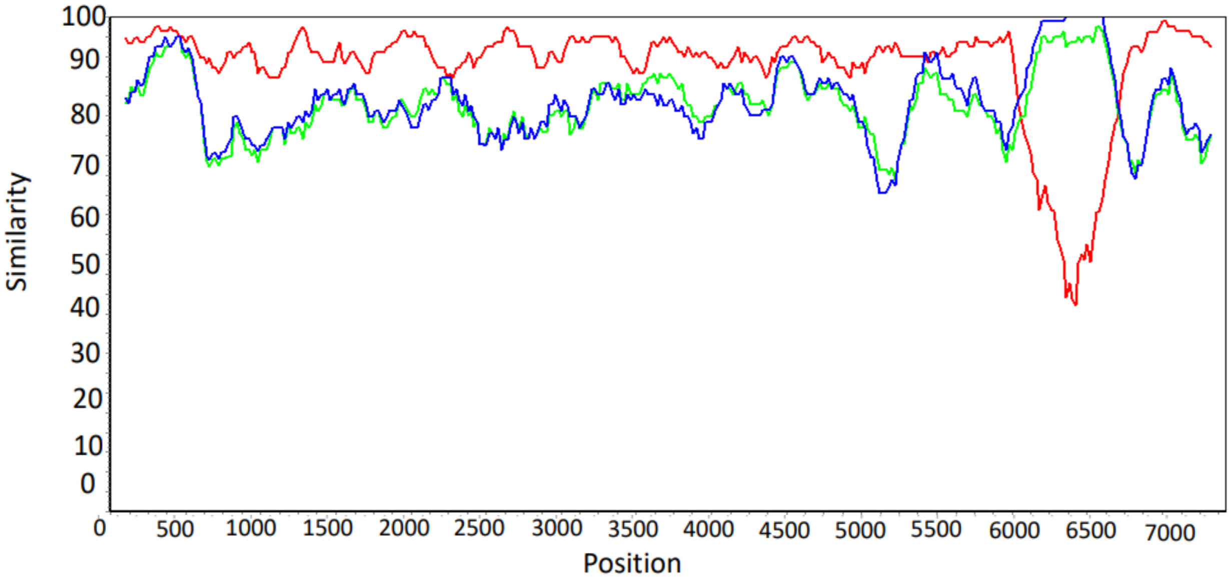

(G) NJ tree based on recombination fragments (6072-7282 nt)

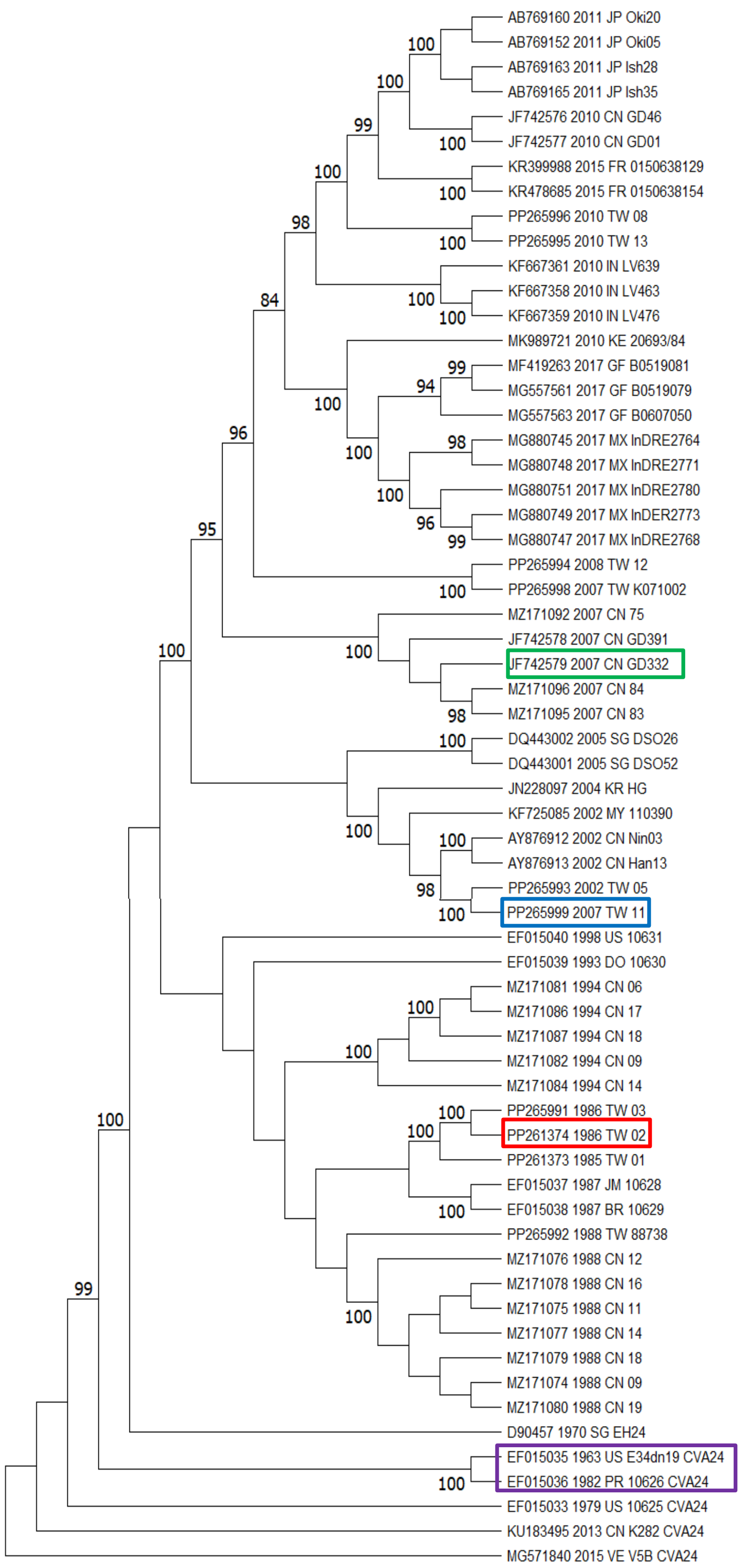

(H) NJ tree based on non-recombination fragments (1-6071, 7283-7422nt)

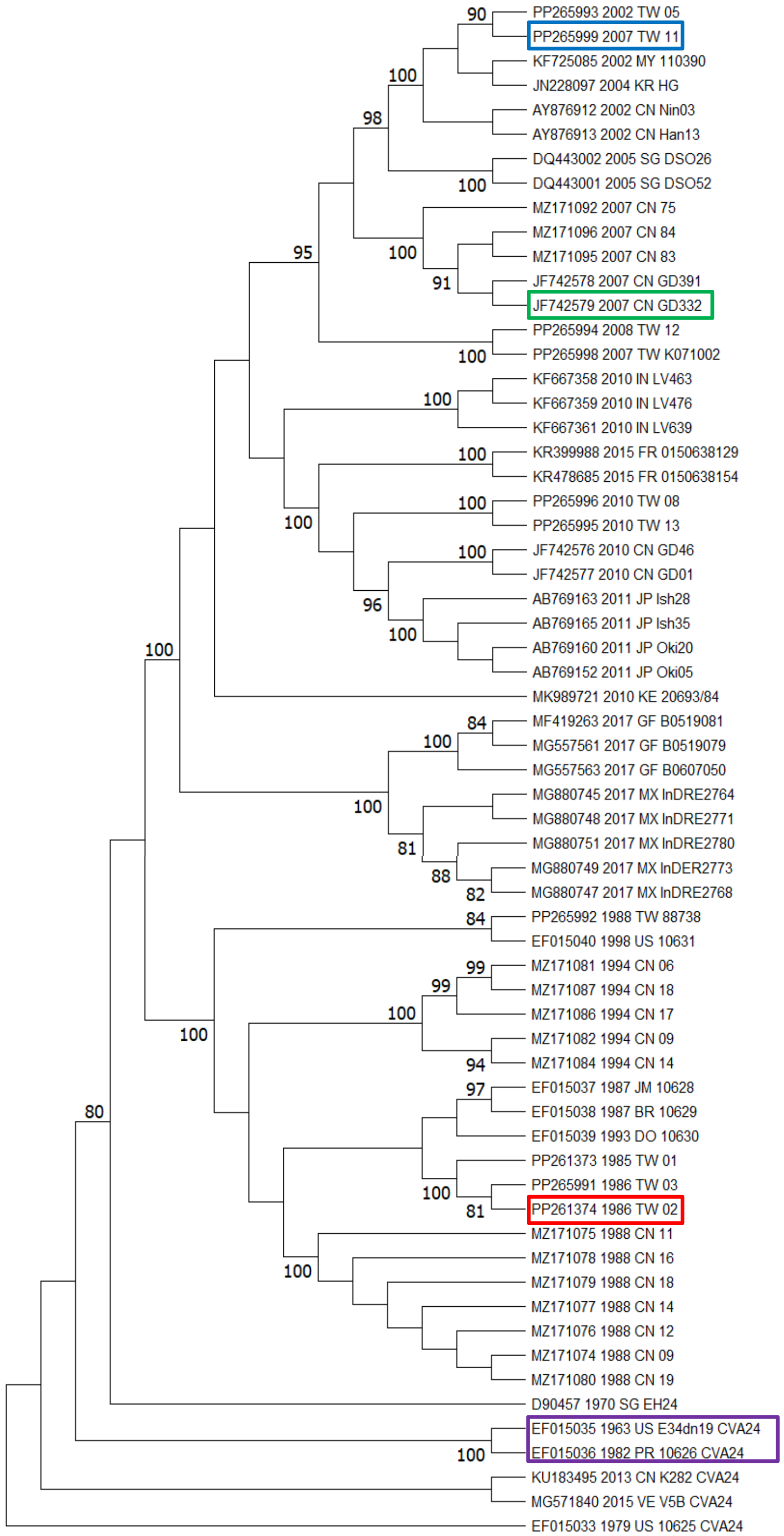

Figure S1 (E) Result of Bootscan Figure S1 (F) Result of SimPlot. Color red, blue, and green represent major parental sequence(s), minor parental sequence(s), and reference parental sequence(s). Figure S1 (G) NJ tree based on recombination fragments (6072-7282 nt) Figure S1 (H) NJ tree based on non-recombination fragments (1-6071, 7283-7422nt). Color red, blue, green, and purple represent major parental sequence(s), minor parental sequence(s), reference parental sequence(s), and recombinant sequence.

CVA24v (EF015037, EF015038)

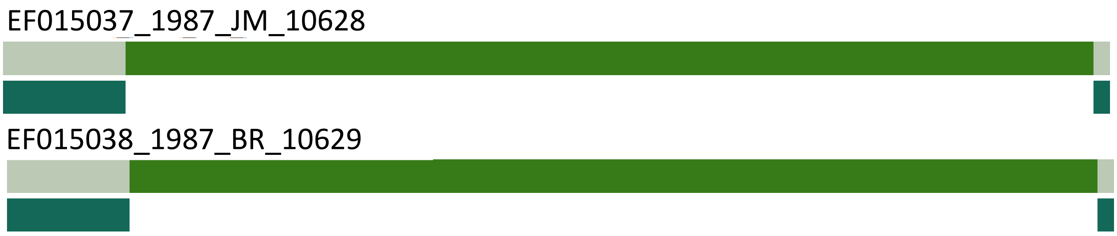

Result of Bootscan

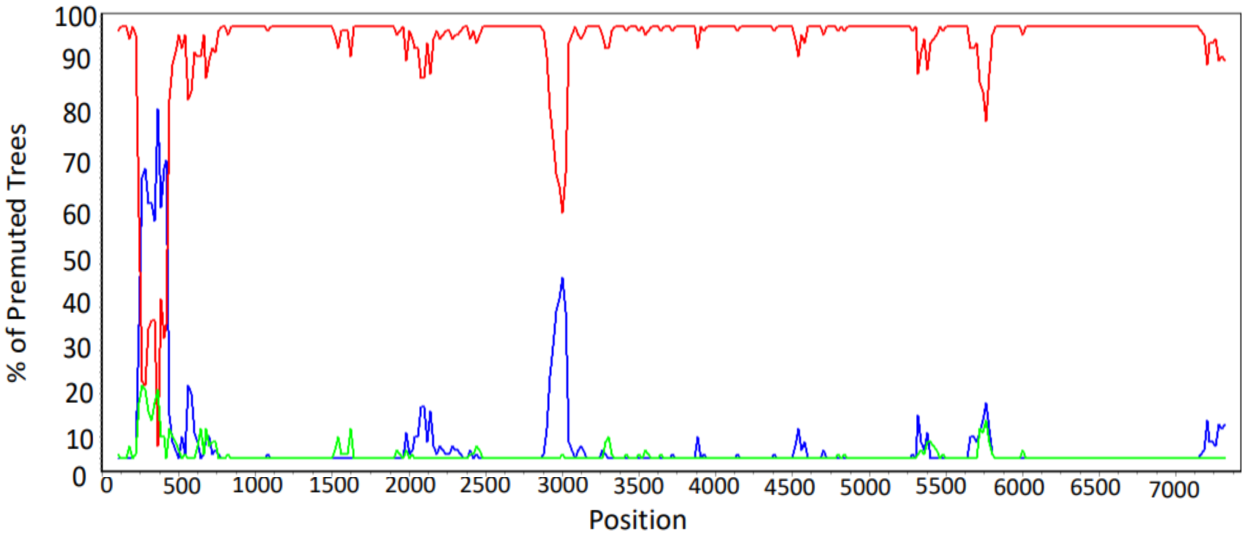

Result of SimPlot

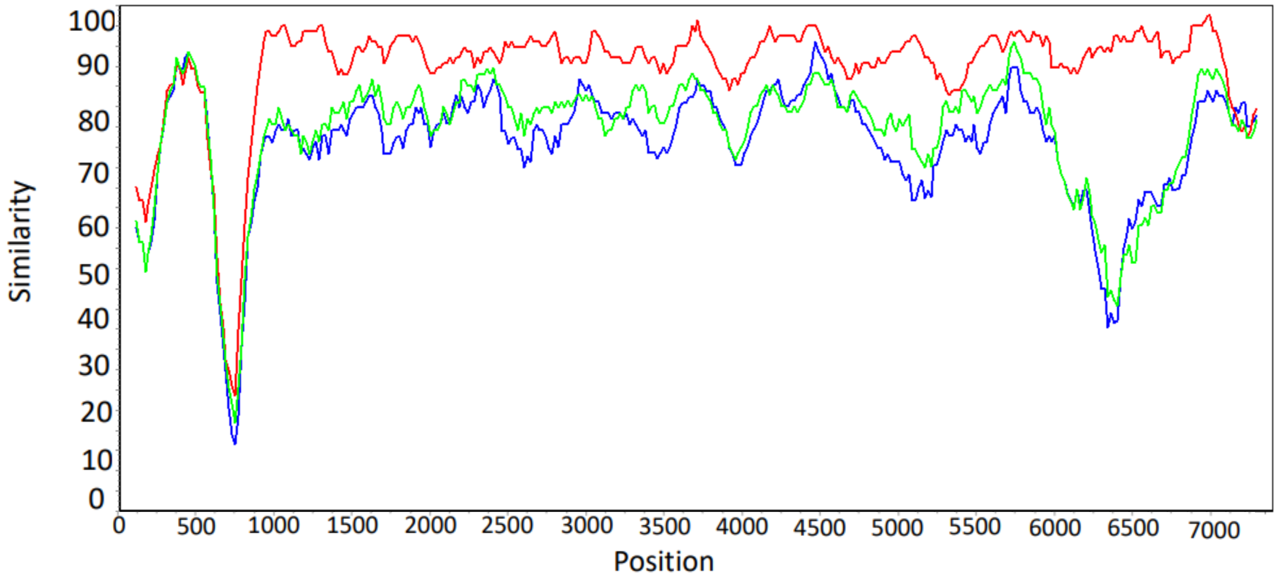

(K) NJ tree based on recombination fragments (1-837, 7305-7422 nt)

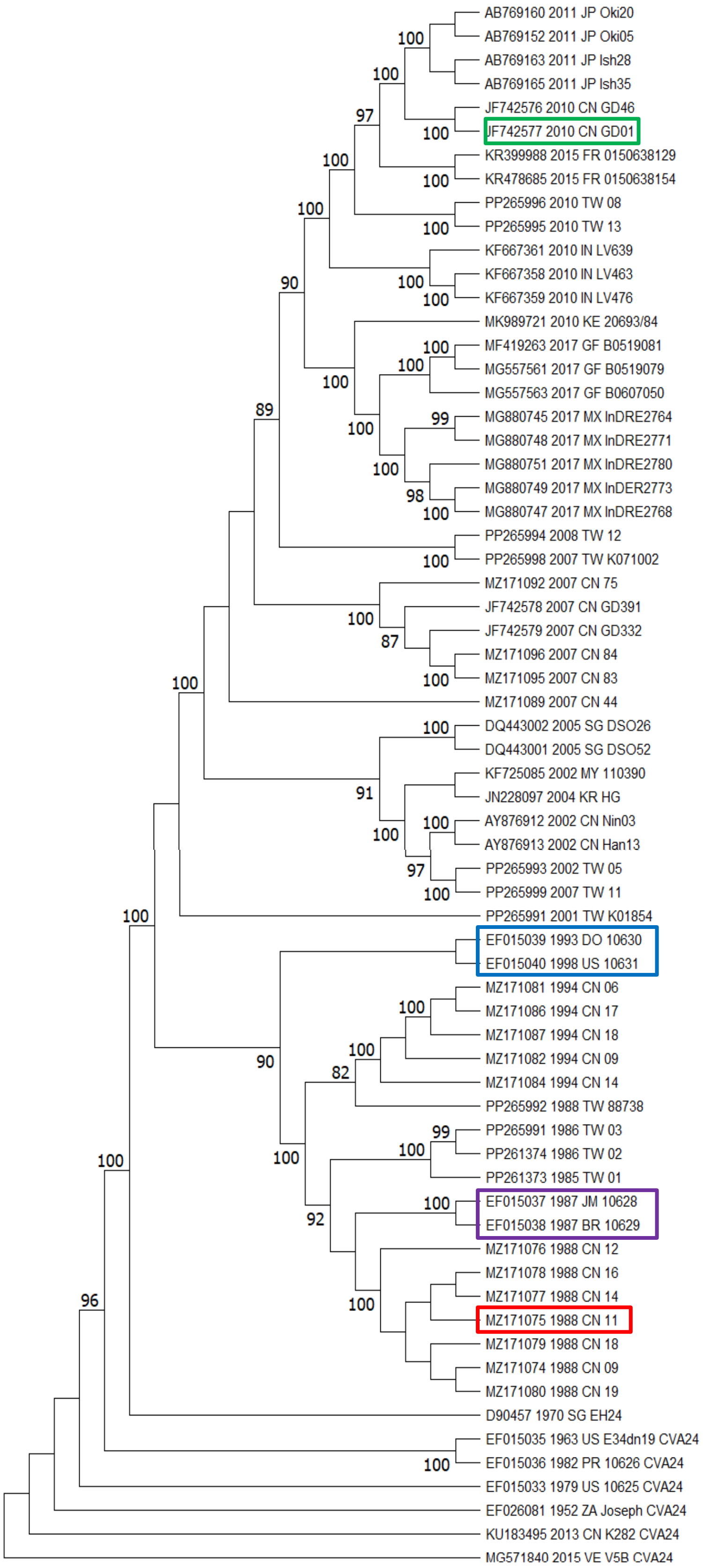

(L) NJ tree based on non-recombination fragments (838-7304nt)

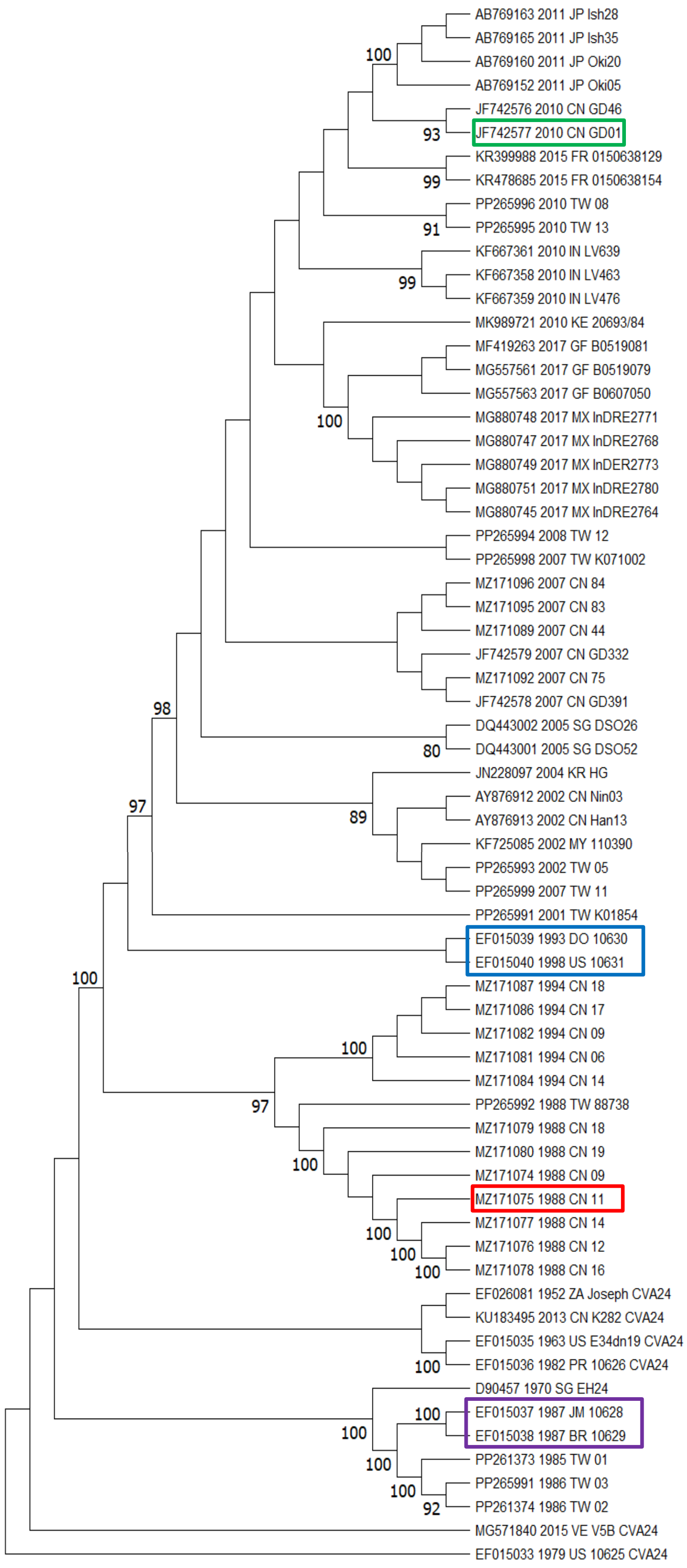

Figure S1 (I) Result of Bootscan Figure S1 (J) Result of SimPlot. Color red, blue, and green represent major parental sequence(s), minor parental sequence(s), and reference parental sequence(s). Figure S1 (K) NJ tree based on recombination fragments (71-837nt) Figure S1 (L) NJ tree based on non-recombination fragments (1-70, 838-7422nt). Color red, blue, green, and purple represent major parental sequence(s), minor parental sequence(s), reference parental sequence(s), and recombinant sequence.

CV-A24v (PP261373, PP261374, PP265991)

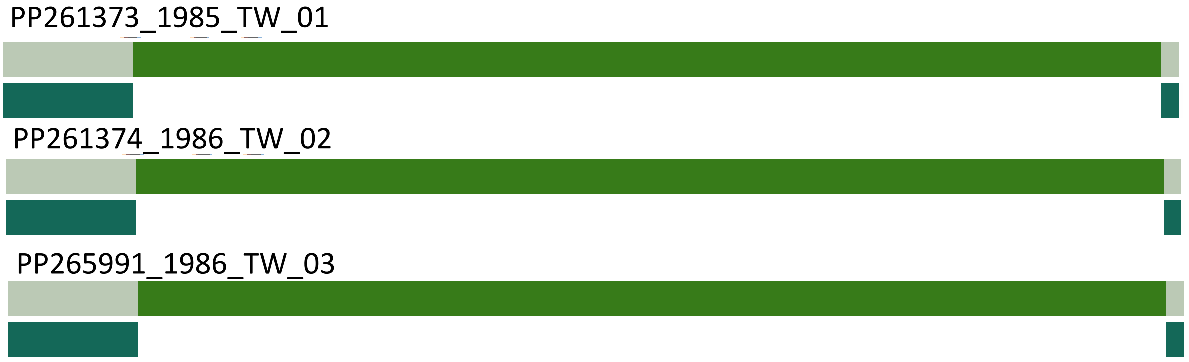

(M) Result of Bootscan

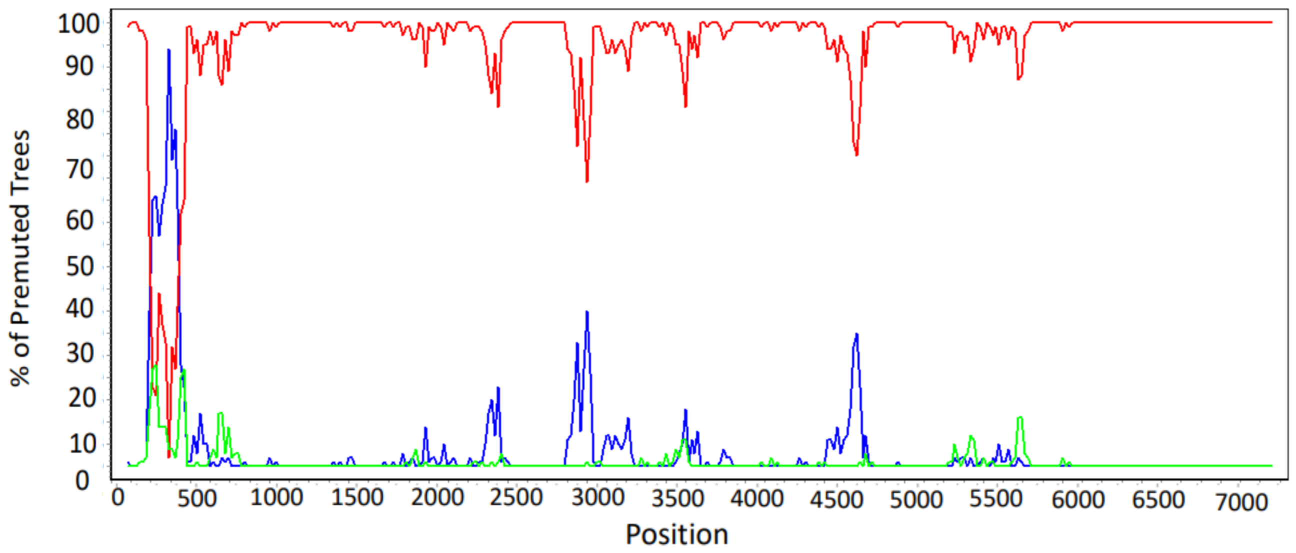

(N)Result of SimPlot

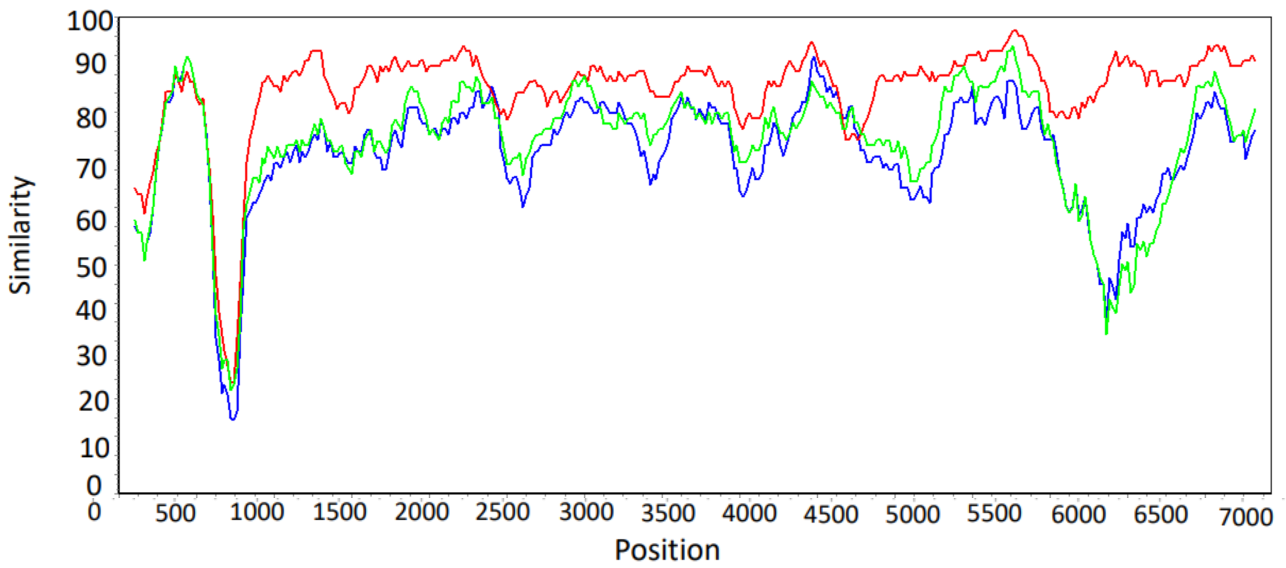

(O) NJ tree based on recombination fragments (1-808nt, 7351-7422nt)

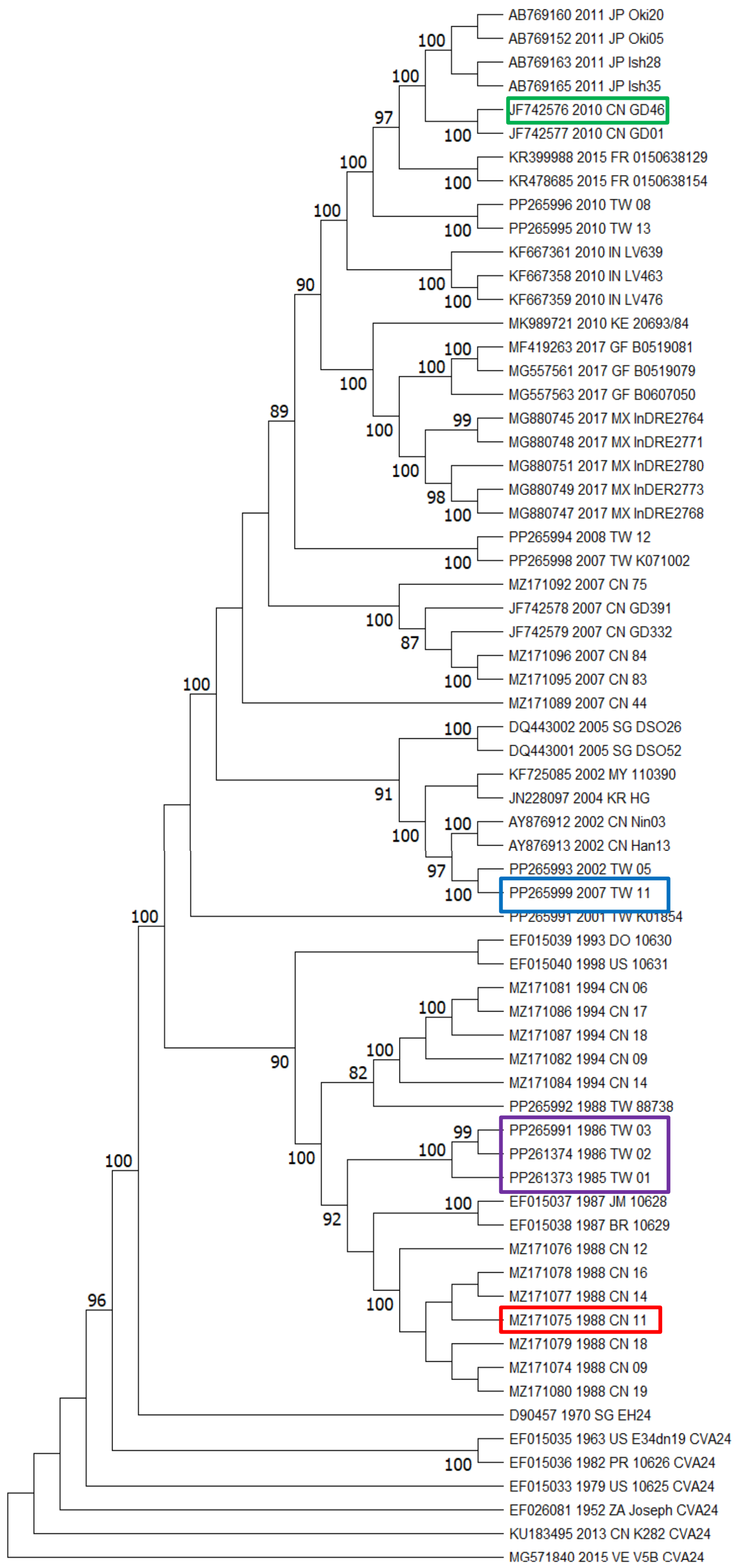

(P) NJ tree based on not-recombination fragments (809-7350 nt)

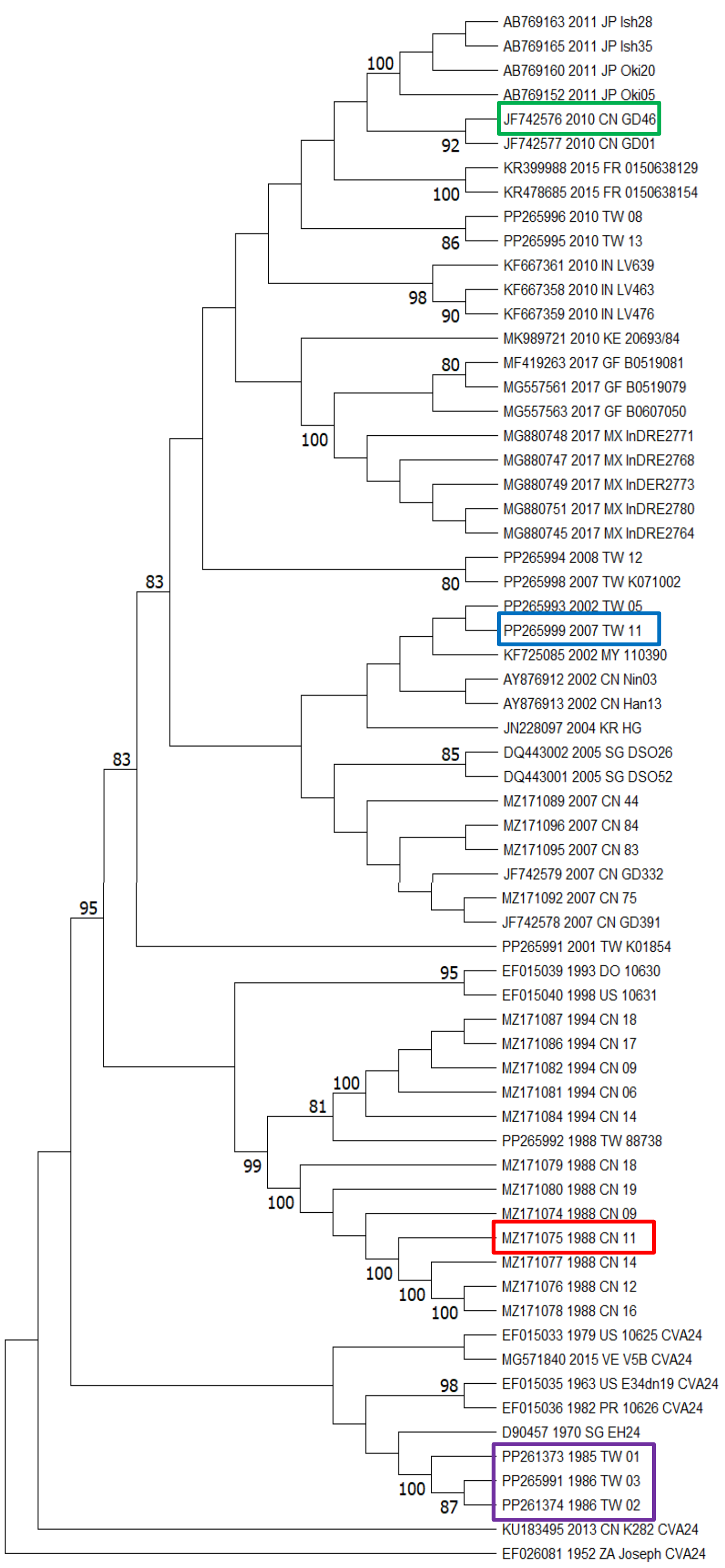

Figure S1 (M) Result of Bootscan Figure S1 (N) Result of SimPlot. Color red, blue, and green represent major parental sequence(s), minor parental sequence(s), and reference parental sequence(s). Figure S1 (O) NJ tree based on recombination fragments (1-808nt, 7351-7422nt) Figure S1 (P) NJ tree based on non-recombination fragments (809-7350 nt). Color red, blue, green, and purple represent major parental sequence(s), minor parental sequence(s), reference parental sequence(s), and recombinant sequence.

CVA24v (EF015039, EF015040)

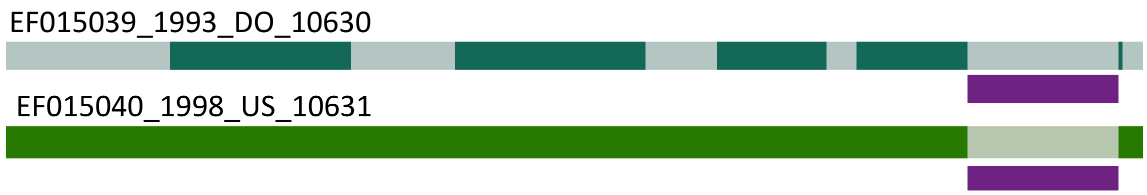

(Q) Result of Bootscan

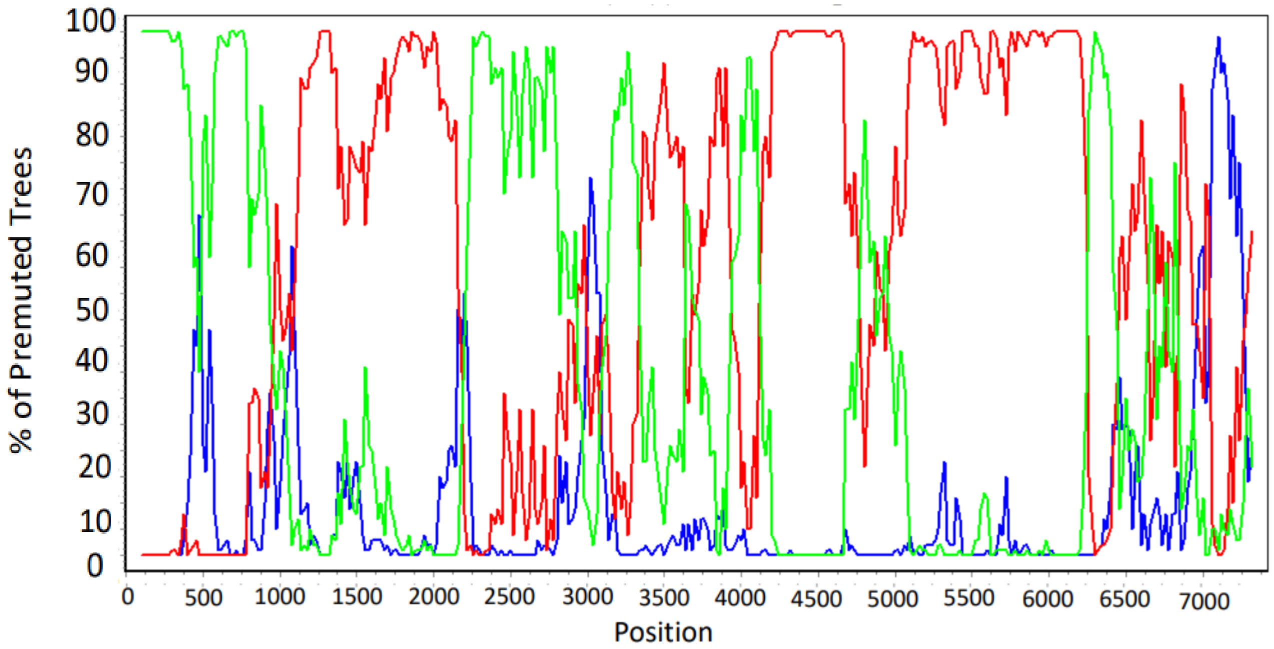

(R)Result of SimPlot

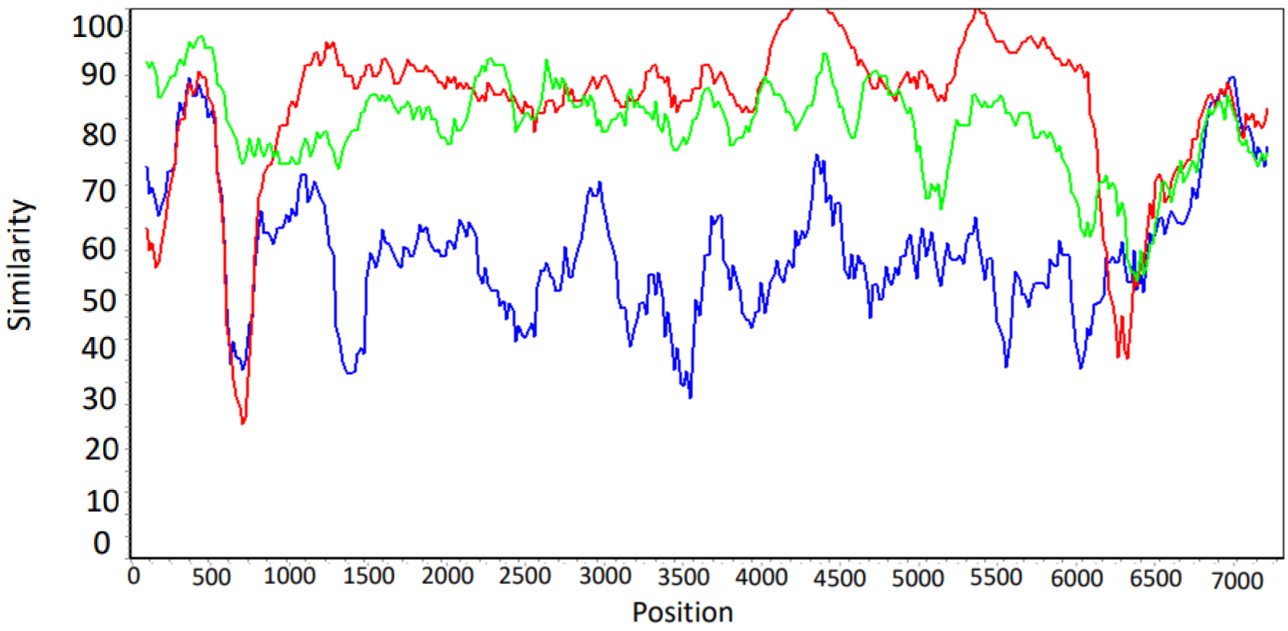

(S) NJ tree based on recombination fragments (6257-7246 nt)

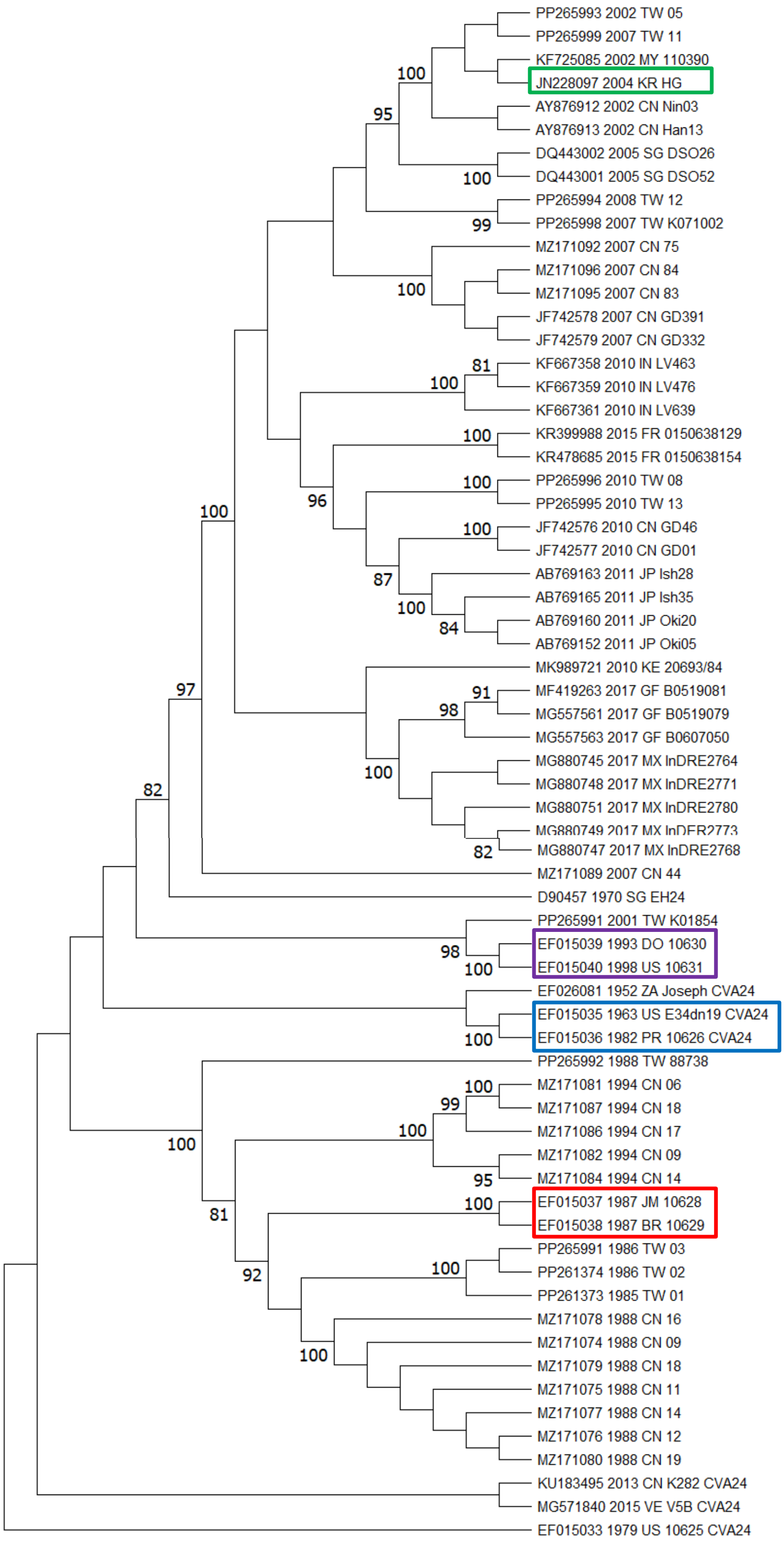

(T) NJ tree based on non-recombination fragments (1-6256nt, 7247-7422nt)

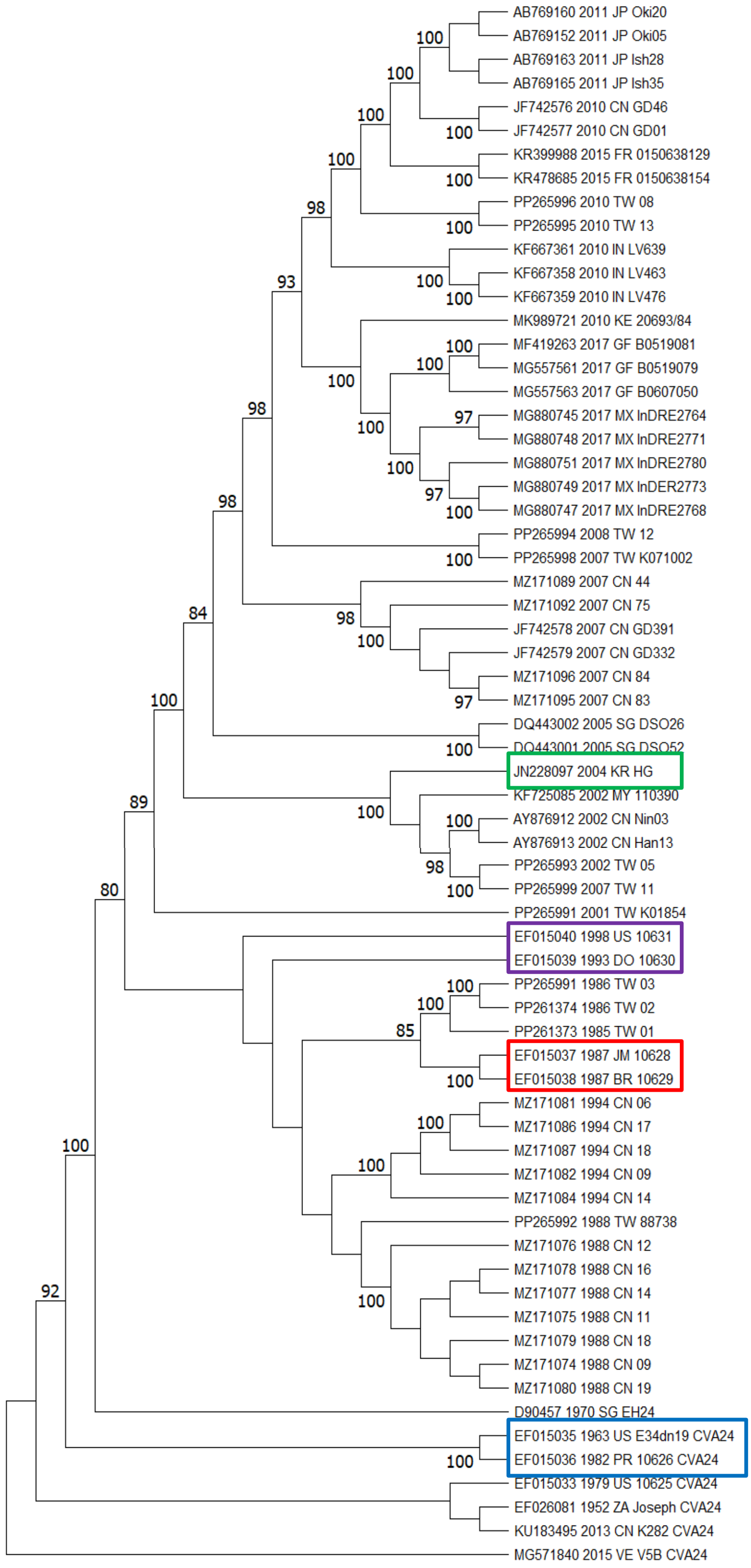

Figure S1 (Q) Result of Bootscan Figure S1 (R) Result of SimPlot. Color red, blue, and green represent major parental sequence(s), minor parental sequence(s), and reference parental sequence(s). Figure S1 (S) NJ tree based on recombination fragments (6257-7246nt) Figure S1 (T) NJ tree based on non-recombination fragments (1-6256nt, 7247-7422nt). Color red, blue, green, and purple represent major parental sequence(s), minor parental sequence(s), reference parental sequence(s), and recombinant sequence.
